# Imidazolinium-Based NHC–Metal Complexes Overcome Both Cancer Multidrug Resistance and Cisplatin Resistance In Vitro

**DOI:** 10.3390/ijms262311382

**Published:** 2025-11-25

**Authors:** Márton Szlávik, Ines Lidia Haffaressas, Réka Mandel, Fanni Fekecs, Ágota Apáti, Attila Paczal, András Kotschy, Gergely Szakács, Szilárd Tóth

**Affiliations:** 1 Servier Research Institute of Medicinal Chemistry, Záhony Utca 7, H-1031 Budapest, Hungary; marton.szlavik.part@servier.com (M.S.); attila.paczal@servier.com (A.P.); andras.kotschy@servier.com (A.K.); 2 Hevesy György PhD School of Chemistry, Eötvös Loránd University, Pázmány Péter Sétány 1/A, H-1117 Budapest, Hungary; 3 Institute of Molecular Life Sciences, HUN-REN Research Centre for Natural Sciences, Magyar Tudósok Körútja 2, H-1117 Budapest, Hungary; haffaressas.ines.lidia@ttk.hu (L.I.H.); rekamandel@gmail.com (R.M.); apati.agota@ttk.hu (Á.A.); szakacs.gergely@ttk.hu (G.S.); 4 Doctoral School of Biology, Institute of Biology, Eötvös Loránd University, Pázmány Péter Sétány 1/C, H-1117 Budapest, Hungary; 5 Center for Cancer Research, Medical University of Vienna, Spitalgasse 23, A-1090 Vienna, Austria; 6 National Laboratory for Drug Research and Development, H-1117 Budapest, Hungary

**Keywords:** N-heterocyclic carbene, multidrug resistance, cisplatin resistance, ABCB1

## Abstract

We report the synthesis and biological characterization of N-heterocyclic carbene (NHC) complexes with gold(I), silver(I), copper(I), and palladium(II) metal centers, and 3-(2,6-diisopropyl-phenyl) imidazolinium- and imidazolium-based ligands, including their biscarbene complexes, along with metal complexes of 4-(S)-tert-butyl-imidazolinium-derived carbenes carrying various substituents in position 1. Compared to the imidazolium complexes, the corresponding imidazolinium complexes displayed superior cytotoxicity against the Mes-Sa uterine sarcoma cell line, while the biscarbene complexes exhibited greatly enhanced cytotoxicity with nanomolar activity. The ABCB1-overexpressing multidrug-resistant sublines of Mes-Sa demonstrated only marginal resistance to monocarbene imidazolinium complexes lacking a 4-(S)-tert-butyl group, whereas significant resistance was observed for all other complexes, with its extent further influenced by the nature of the metal center. Probing a subset of the complexes confirmed their strong cytotoxicity against the CST murine breast cancer cell line and its cisplatin-resistant variant, with little or no cross-resistance observed. Within a defined subset, compounds triggered apoptosis, and intracellular ROS production was consistently induced by the copper complexes. Collectively, these results indicate that imidazolinium-based metal NHCs are promising anticancer drug candidates, with copper and silver centers standing out for their potent cytotoxicity and evasion of both ABCB1-mediated and cisplatin resistance.

## 1. Introduction

The widespread use and proven effectiveness of cisplatin in cancer therapy has inspired the development of metal-containing anticancer agents [1,2]. In this context, N-heterocyclic carbene (NHC)–metal complexes of imidazolium salts are of great interest, owing to their ability to be “fine-tuned” through the choice of metal center and the virtually unlimited possibilities for decorating the heterocyclic ring with diverse substituents [3,4]. Moreover, the imidazole ring is a structural motif found in numerous anticancer agents, both approved and in the drug research pipeline [5], further proving the prominence of imidazoles among promising drug candidates.

Transition metal complexes of imidazolium type NHCs, such as silver, gold, copper, and palladium complexes exert cytotoxicity against a wide range of cancer cells in vitro, often at submicromolar concentrations [3,4,6,7,8,9]. Cancer drug resistance, however, can undermine the effectiveness of these drugs, as cross-resistance mechanisms may reduce their cytotoxicity by several fold [6,8,10].

Overexpression of ABCB1 (P-glycoprotein) is a common resistance mechanism that can be induced by a wide range of chemotherapeutic drugs, such as anthracyclines, vinca-alkaloids, or taxanes, resulting in multidrug resistance (MDR) [11]. A classical example is the Mes-Sa/Dx5 cell line, which developed stable ABCB1 overexpression and resistance after continuous selection with doxorubicin, and is widely used as an in vitro model of MDR [12]. On the other hand, resistance to cisplatin is mediated by mechanisms unrelated to ABCB1, which can nevertheless confer broad cross-resistance [13]. For example, restoration of homologous recombination by backmutation in BRCA1/2-mutant cancers abrogates sensitivity to cisplatin and other DNA crosslinking agents [14].

To date, no comprehensive structure–activity relationship has been established regarding the cytotoxicity and resistance profiles of imidazolinium-based NHC-metal complexes. In this study, we synthesized imidazolinium and imidazolium complexes with copper(I), silver(I), gold(I) and palladium(II) centers to investigate their anticancer activity against both parental and drug-resistant cell lines. These metals—especially gold(I) and silver(I)—are among the most frequently investigated centers for NHC complexes, not only for classical imidazolium-type ligands but also for broader NHC classes such as benzimidazoles and triazoles [15,16,17,18,19,20,21]. Therefore, examining their behavior in resistance models is essential for understanding the contribution of the metal center to cytotoxicity and drug resistance, and accordingly, it is aimed at frequently in Au(I)-NHC development [22]. Our series included novel 4-(*S*)-*tert*-butyl-imidazolinium complexes, which have not been previously tested against cancer cells, and biscarbene complexes, which are known to exert increased cytotoxicity compared to monocarbene complexes [23]. The cytotoxicity of all compounds was tested in an in vitro triple coculture model system for ABCB1-mediated MDR in uterine sarcoma lines, and a subset was probed against a triple-negative, BRCA1-deficient murine breast cancer line and its cisplatin-resistant derivative.

## 2. Results

### 2.1. Structure and Synthesis of the Tested Compounds

**Figure 1:**
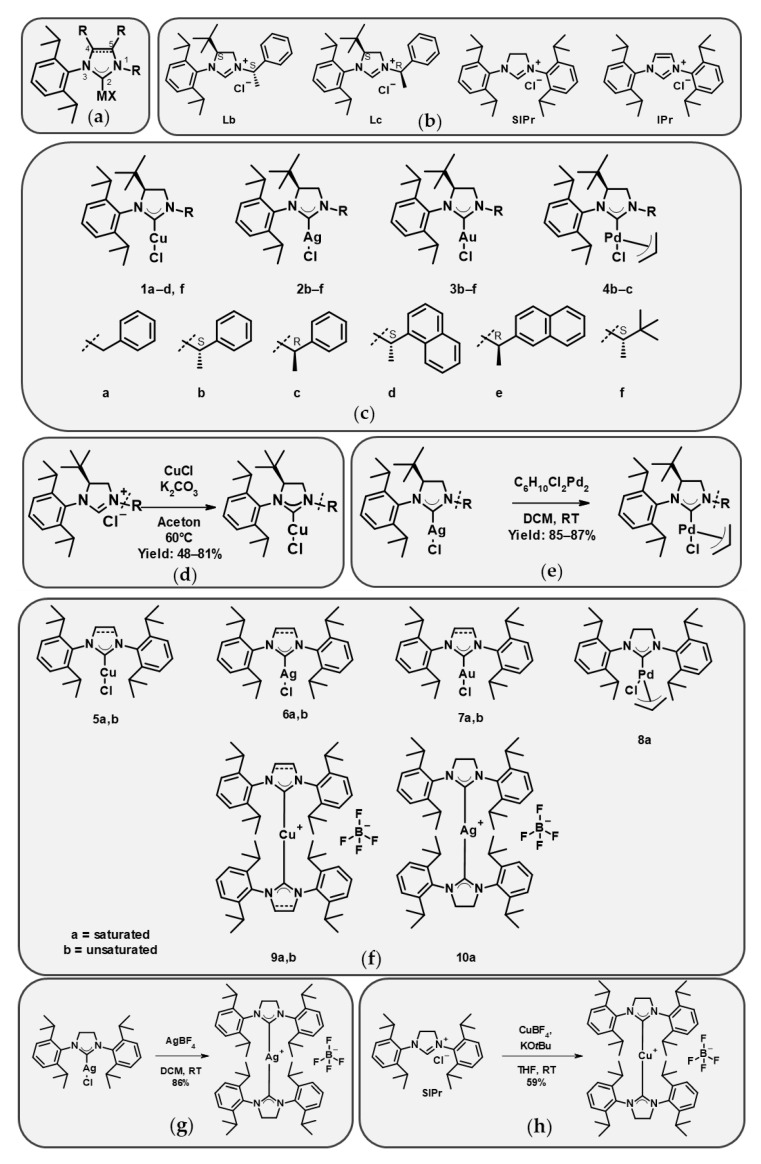
The structures of the investigated compounds. (**a**) The general structure and numbering of the positions. X represents chlorine in monocarbenes. (**b**) The structures of the tested free ligands. (**c**) The structures of the 4-(*S*)-*tert*-butyl analogues, where “**a**–**f**” represent R in position 1. (**d**) The general synthesis of copper complexes **1a**–**d**, **f**. (**e**) The general synthesis of palladium complexes **4b**,**c**. (**f**) The structures of the tested SIPr- (“**a**”, saturated) and IPr-based (“**b**”, unsaturated) mono- and biscarbene complexes. (**g**) Synthesis of silver biscarbene **10a** and (**h**) copper biscarbene **9a**.

Figure 1a compiles the set of imidazolium and imidazolinium salts that were used as carbene precursors and the derived metal complexes. Some of the NHC precursors are commercially available (**SIPr**, **IPr**, **5a**,**b**, **6a**,**b**, **7a**,**b**, **8a**, and **9b**), while others were synthesized and are described here (**4b**,**c**, **9a**, **10a**) or have previously been described (**Lb**, **Lc**, **1a**–**d**,**f**, **2b**–**f**, **3b**–**f** [24], and **1a**–**d**,**f** [Szlávik et al., under preparation]). The structures are shown in Figure 1b,c,f; the synthesis of the copper complexes is shown in Figure 1d; and the synthesis of the compounds introduced here is described in Figure 1e,g,h. Substituents on the ligand were introduced at positions 1, 3, and 4 (for clarity, the numbering follows the general formula in Figure 1a). Several of the analogues have a chiral center in position 1, and both *R* and *S* enantiomers were included in the set, enabling the evaluation of stereochemical effects.

The whole set used in this study consisted of nine copper(I), seven silver(I), seven gold(I), and three palladium(II) monocarbene complexes, as well as two copper(I) and one silver(I) biscarbene complexes, with partly overlapping sets of ligands, and four ligand precursors.

### 2.2. Cytotoxicity of Compounds Against the Mes-Sa Uterine Sarcoma Cell Line

**Figure 2:**
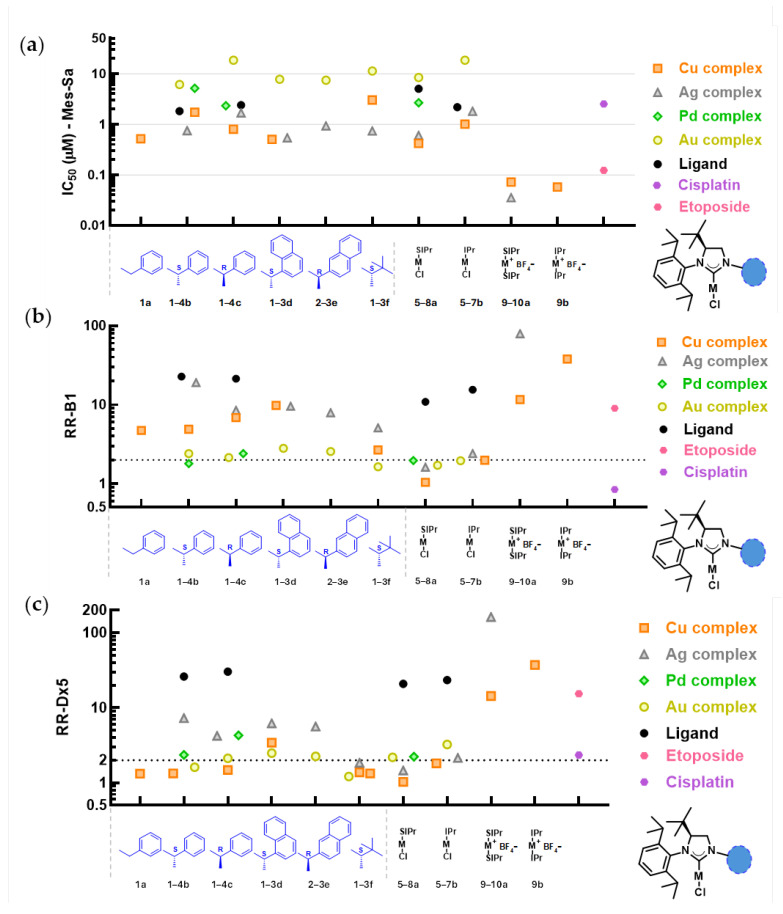
(**a**) Cytotoxicity of the metal-free ligands, mono- and biscarbene complexes of copper(I), silver(I), palladium(II), and gold(I), and control compounds etoposide and cisplatin expressed as IC_50_ [µM] values against the parental uterine sarcoma cell line Mes-Sa mCherry. (**b**,**c**) Resistance ratio (RR, IC_50___MDR_/IC_50___parental_) of the MDR variants Mes-Sa/B1 mOrange (RR-B1) and Mes-Sa/Dx5 eGFP (RR-Dx5). Dashed lines are drawn at the RR value of 2, indicating 2-fold resistance. *S*/*R*: configuration on the methylene carbon in position 1. Groups in position 1 of the 4-(*S*)-*tert*-butyl monocarbenes are drawn in blue. M represents the metal center, Cl represents chlorine, and BF_4_^−^ stands for tetrafluoroborate.

All synthesized complexes and representatives of the respective ligands (**Lb**, **Lc**, **SIPr**, **IPr**) were tested in a triple coculture of uterine sarcoma cell lines, consisting of the parental Mes-Sa cell line, and its MDR derivatives Mes-Sa/B1 and Mes-Sa/Dx5, transfected or selected in doxorubicin to overexpress P-glycoprotein, respectively (Figure 2a–c, Table S2). Each cell line expresses a distinct fluorescent protein (mCherry, mOrange, and eGFP, respectively), and the total fluorescence of a well is proportional to the cell number [25], allowing the assessment of compound cytotoxicity.

The metal-free ligands exhibited relatively mild cytotoxicity against the parental line Mes-Sa (IC_50_ values between 1.83 and 5.05 µM). Cytotoxicity increased upon complexation with Cu(I) or Ag(I), whereas Au(I) complexation led to a decrease. Accordingly, across the entire set, Cu(I) and Ag(I) complexes were generally more cytotoxic than Au(I) complexes, with Pd(II) complexes showing intermediate toxicity. 4-(*S*)*-t*Bu substituted copper complexes with aromatic moieties at position 1 (**1a**–**1d**) and the imidazolinium and imidazolium complexes with IPr and SIPr (**5a**,**b**) exerted remarkable cytotoxicity (IC_50_ values: 0.42–1.74 µM), while the 1-alkyl-substituted complex **1f** was slightly less cytotoxic (3.04 µM). 4-(*S*)*-t*Bu imidazolinium and SIPr and IPr silver complexes possessed similar cytotoxicity to copper complexes (0.54–1.82 µM), regardless of whether they had 1-alkyl or 1-aryl moieties. The palladium complexes’ cytotoxicity was in the low micromolar range (2.34–5.19 µM), while the cytotoxicity of gold complexes **3b**–**3f** was one order of magnitude lower (IC_50_ values between 6.2 and 18.6 µM) than that of the copper- and silver-centered analogues. Biscarbene complexes of copper and silver were one order of magnitude more cytotoxic than the respective monocarbene complexes (0.036–0.072 µM).

### 2.3. Cytotoxicity Against MDR Cancer Cells

The cytotoxicity pattern against the resistant cell lines differed from that observed in Mes-Sa, and was quantified as the resistant ratio (RR, IC_50___MDR_/IC_50___parental_). Mes-Sa/B1 displayed strong resistance to the metal-free ligands (RR: 10.9–22.7). For monocarbene complexes, considerable resistance was observed in the case of the copper- and silver-centered 4-(*S*)-*t*Bu complexes (RR: 2.7–9.8 and RR: 5.1–19.1, respectively), whereas against IPr-based complexes of the same metals, resistance was marginal (RR: 2.0 and 2.4, respectively) and SIPr-based complexes evaded ABCB1-mediated resistance (RR: 1.0 and 1.6, respectively). For gold and palladium complexes, resistance was low to marginal across the entire set, including 4-(*S*)-*t*Bu, SIPr, and IPr analogues (RR: 1.6–2.8 and 1.8–2.4, respectively). The biscarbene complexes of copper and silver triggered marked resistance (RR: 11.6–38.0 and 79.5, respectively). In comparison, Mes-Sa/B1 elicited resistance against the known ABCB1 substrate chemotherapeutic agent etoposide with an RR of 9.0.

Mes-Sa/Dx5 has higher ABCB1 expression than Mes-Sa/B1, resulting in greater resistance against ABCB1-substrates [25]. Accordingly, against etoposide, the RR of Mes-Sa/Dx5 was 15.4, and the RR values were between 20.9 and 30.4 against the metal-free salts (**Lb**, **Lc**, **SIPr**, **IPr**). Despite the higher ABCB1 expression levels, the resistance of Mes-Sa/Dx5 against monocarbene copper complexes was marginal (RR: 1.0–3.4); against silver complexes, resistance was low to moderate (RR: 1.5–7.3); and against gold complexes, resistance was low to marginal (RR: 1.2–3.2); and lastly, against palladium complexes, we observed 2.2–4.3-fold resistance. Against the biscarbene complexes of copper, the RR values of Mes-Sa/Dx5 were 14.3 and 37.3 for **9a** and **9b**, respectively, while for the silver centered **10a**, the resistance was extremely high (RR: 162.2).

### 2.4. Cytotoxicity of Compounds Against a Murine Breast Cancer Cell Line and Its Cisplatin-Resistant Derivative

**Figure 3:**
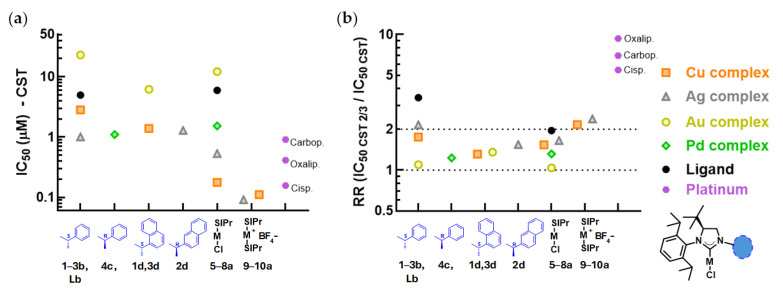
(**a**) Cytotoxicity of the selected ligands, metal complexes, and platinum drugs cisplatin, carboplatin, and oxaliplatin against the murine breast cancer cell line CST. (**b**) The resistance ratio (RR) of the CST-2/3 cell line against the tested compounds. Dashed lines are drawn at RR values of 1 and 2, indicating the interval of the absence of significant resistance. *S*/*R*: configuration on the methylene carbon in position 1. Groups in position 1 of the 4-(*S*)-*tert*-butyl monocarbenes are drawn in blue. M represents the metal center, Cl represents chlorine, and BF_4_^−^ stands for tetrafluoroborate.

A set of the ligands and metal complexes was tested against the BRCA-deficient murine breast cancer line CST, and its cisplatin-resistant derivative CST-2/3 (Figure 3, Table S3). Similarly to the results obtained with the parental Mes-Sa line, monocarbene copper and silver complexes were more cytotoxic against parental CST (0.18–2.81 µM and 0.53–1.28 µM, respectively) than the free ligands **Lb** and **SIPR** (4.95–5.92 µM). Palladium complexes also displayed higher cytotoxicity compared to their respective ligands (1.10 and 1.52 µM), while gold complexes showed only moderate cytotoxicity (6.2–22.9 µM). The biscarbene complexes of copper and silver were the most cytotoxic (**9a**, 0.11 µM, and **10a**, 0.092 µM). As controls, carboplatin, oxaliplatin, and cisplatin were used, all showing submicromolar IC_50_ values (0.15–0.89 µM). CST 2/3 cells were resistant against the platinum control drugs (RR: 5.5–9.3), whereas resistance was marginal or absent for all of the tested mono- and biscarbene complexes (RR: 1.0–2.4). Interestingly, the resistance against the free ligands was greater (RR: 2.0–3.4).

### 2.5. Cytotoxicity Against a Mesenchymal Stem Cell-like Cell Line

We assessed the cytotoxicity of some selected complexes against a non-malignant cell line, the mesenchymal stem cell-like MSCL-1 line, which was generated from human embryonic stem cells [26]. Compared to the parental lines Mes-Sa and CST (Table S4), monocarbene complexes **5a**, **6a**, and **7a** were between 2.1- and 6.5-fold less active against MSCL-1, while the Pd(II)-centered **8a** was as cytotoxic to MSCL-1 as to Mes-Sa, and only marginally less active as it was for CST (1.1 and 2.0-fold, respectively). Biscarbene complexes **9a** and **10a**, however, possessed stronger, 6.1–15.7-fold selectivity against the cancer cells that exceeded the selectivity of cisplatin against Mes-Sa (6.8-fold). Against CST, cisplatin selectivity was 113.6-fold compared to MSCL-1, which accounted for the loss of BRCA1 function of this cancer line, making it hypersensitive to DNA-damaging agents. The selectivity of the free SIPR ligand against Mes-Sa and CST was 2.3- and 2.0-fold, respectively.

### 2.6. Intracellular ROS Generation

**Figure 4:**
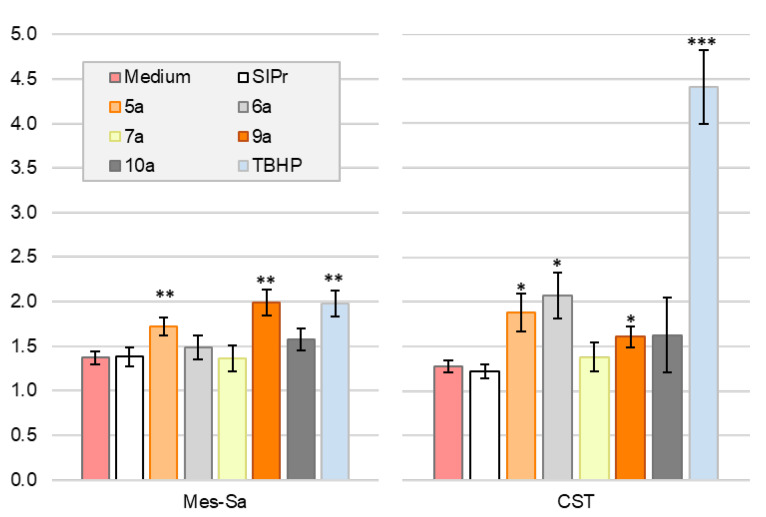
Intracellular ROS induction measured by the H_2_DCFDA assay for the imidazolinium salt **SIPr** and its monocarbene complexes, **5a**, **6a**, and **7a,** and biscarbene complexes **9a** and **10a** in Mes-Sa and CST cell lines at 25 µM concentration during 4h incubation. The scale represents a fold change in the fluorescent signal compared to 0 h. Additionally, 25 µM *tert*-butyl hydroperoxide (TBHP) served as a positive control. *, *p* < 0.1; **, *p* < 0.05; ***, *p* < 0.01.

For mechanistic investigations, we selected mono- and biscarbene metal complexes of the imidazolinium salt SIPr. The H_2_DCFDA assay was performed to assess whether the compounds induced intracellular reactive oxygen species (ROS) in Mes-Sa and CST cell lines (Figure 4). In Mes-Sa, after 4 h of incubation, silver and gold complexes **6a**, **7a**, **10a**, and **SIPr** did not significantly induce intracellular ROS production compared to serum-containing medium. In contrast, the copper complexes **5a** and **9a** triggered a 72% and 99% increase in ROS, respectively, that was significantly higher than the 37% increase observed in the serum-containing medium. In CST cells, ROS levels were significantly increased by copper complexes **5a** (88%) and **9a** (61%), as well as by the silver complex **6a** (by 107%), compared to the untreated control, which showed a 28% increase over 4 h. No significant ROS induction was observed for **SIPr**, **7a**, and **10a**.

### 2.7. Apoptosis—Annexin V and Propidium Iodide Staining

**Figure 5:**
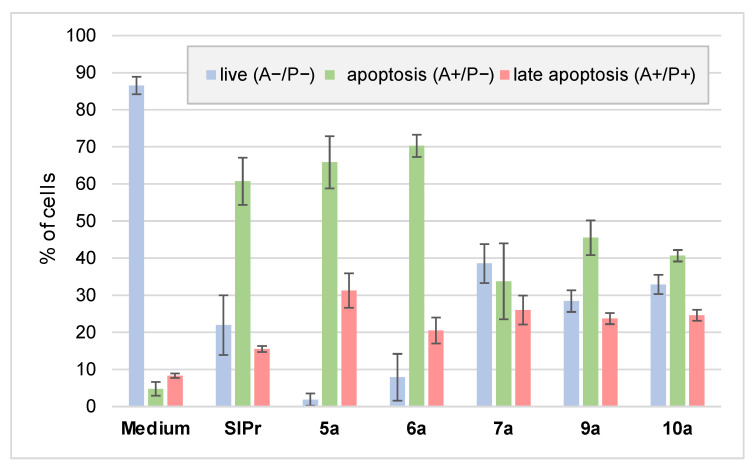
Proportion of cells in late and early apoptosis. A: Annexin V; P: propidium iodide staining.

We evaluated whether the complexes can trigger apoptosis, the preferred mode of cell death for cytotoxic agents. Mes-Sa cells were treated with the respective analogues for 72 h, followed by Annexin V and propidium iodide staining. All tested compounds, including the metal-free ligand, increased the proportion of both early and late apoptotic cells (Figure 5). Among them, the gold complex and the biscarbene complexes induced the highest proportion of late apoptotic cells.

## 3. Discussion

Imidazolinium-based NHCs with various substitutions, especially in positions 1 and 3, and with various metal centers, have been widely investigated for their in vitro and in vivo anticancer activity, with promising results [27,28,29,30]. In this study, we identified imidazolinium-based NHC complexes with 3-diisopropyl-phenyl moiety and with various substitutions in position 1, with or without 4-(*S*)-*tert*-butyl group, as potent anticancer agents, with several prominent analogues retaining their activity in two clinically relevant resistance settings, in ABCB1-mediated multidrug resistance and cisplatin resistance models.

Cytotoxic potency was fundamentally influenced by the nature of the metal center. Cu(I)- and Ag(I)-centered complexes were superior to Pd(II) complexes, while Au(I) complexes were only slightly cytotoxic. Biscarbenes were an order of magnitude more cytotoxic than the respective monocarbenes; however, this came at the cost of significant ABCB1-mediated resistance. While decoration of the imidazol(in)ium ring in positions 1 and 4 did not cause dramatic differences in cytotoxicity, saturated SIPr-based complexes were 2–3-fold more cytotoxic compared to matching unsaturated IPr-based complexes.

Stereochemistry in position 1 had only a minimal, context-dependent effect. The *R* configuration of the enantiomers of the 1-(1′-methyl)-benzyl metal complexes (**1b**,**c**–**4b**,**c**) were only slightly more cytotoxic compared to the *S* configuration, when Cu(I) or Pd(II) were the metal centers (**1b** vs. **1c**, and **4b** vs. **4c**), and less cytotoxic when it was Ag(I) or Au(I) (**2b** vs. **2c**, and **3b** vs. **3c**). However, we did not observe any cytotoxicity trends between the Ag(I) and Au(I) complexes **2**–**3d** and **2**–**3e**, and the Cu(I) complexes 1b* and 1c* were equipotent (Table S2, Figure S2). Taken together, steric differences in position 1 did not play an important role in the molecular interactions behind cytotoxicity or resistance. A low impact of steric effects was also found in a study of 1,3-dicarboxylate-type imidazolium NHCs with silver centers, where the enantiomer with the configuration of (*R*)-isopropyl was 1.3–2.6 times more cytotoxic in a panel of five cell lines, compared to the *S* configuration, while (*R*)- and (*S*)-methyl analogues were equipotent [31].

Using the triple coculture MDR model, we demonstrated that several monocarbenes, especially SIPr-based complexes, overcame ABCB1-mediated resistance, whereas we found significant resistance against biscarbenes, which is consistent with increased size and hydrophobicity favoring recognition by this transporter [32]. In addition to carbene multiplicity, resistance was governed by the metal center. Cu(I) and Ag(I) complexes of 4-(*S*)-*t*Bu analogues acted as more avid ABCB1 substrates than Pd(II) or Au(I) complexes. In contrast, we did not observe remarkable cross-resistance to either mono- or biscarbenes on the cisplatin-resistant cell line CST-2/3; thus, modulation of DNA-repair mechanisms providing cross-resistance against DNA-damaging agents does not grant rescue for the cancer cells against the selected NHC complexes, and thus presumably DNA is not a primary target of the analogues.

According to a comprehensive review [33], it is common for NHC complexes to trigger apoptosis by inducing endoplasmic reticulum stress or intracellular ROS, or targeting members of the Bcl-2 family proteins, p53, cytochrome c, or thioredoxin reductases (TrxR). Although the detailed mechanism of action of the NHC complexes is not fully understood, docking studies suggest that they bind to their intracellular target protein as an intact complex, or to, e.g., cysteine or serine residues by ligand exchange, releasing the chlorine from the complex.

In our mechanistical studies, the selected SIPr-based mono- and biscarbene copper complexes triggered intracellular ROS generation and apoptosis consistently, suggesting a redox active copper-driven cell death pathway, presumably through Fenton-like reactions [34], similarly to our previous observation with the copper(I) complex Cu-LA [8], which is a monocarbene complex of the imidazolinium-based natural NHC called lepidiline A (LA). In line with the results of Ag-LA and Au-LA [8], selected SIPr-based Ag(I) and Au(I) complexes did not induce a general ROS production in the tested cell lines. According to a study, the silver complexes that are denoted in the present study as **6a** and **6b** triggered apoptosis by targeting the mitochondria [6], while structurally similar silver and gold NHC complexes inhibited the mitochondrial TrxR [35]; therefore, the presented NHC complexes might induce cell death via TrxR inhibition.

Finally, except for the Pd(II) complex **8a**, the tested compounds were more cytotoxic to the parental cancer cell lines Mes-Sa and CST than against the non-malignant MSCL-1 cell line. This is a promising result, as in our previous study, the Cu(I)- and Ag(I)-centered lepidiline A-based complexes were not consistently more or less active against cancer cells than against bone marrow or adipose tissue-derived mesenchymal stem cells, while the Au(I) complex was more cytotoxic to the stem cells. In other studies, imidazolium-derived silver NHCs were also lacking a clear trend regarding the cytotoxicity against cancerous cell lines and epithelial cells or fibroblasts [36,37]; thus, more detailed investigations on relevant non-cancerous models are needed in future development.

## 4. Materials and Methods

Materials and reagents, unless otherwise indicated, are commercially available and were used without further purification. Etoposide, cisplatin, and carboplatin were sourced from Accord Healthcare, oxaliplatin, and the ligands **SIPr** and **IPr**, and their monocarbene complexes **5a**,**b**, **6a**,**b**, **7a**,**b**, and **8a**, and the biscarbene **9b** was purchased from Sigma-Aldrich (Merck KGaA, Darmstadt, Germany).

### 4.1. General Synthetic Methods

The reactions were monitored by GC-MS, HPLC-MS, and NMR measurements. For the GC-MS measurements, an Agilent 6850 gas chromatograph (15 m × 0.25 mm column, 0.25 mm HP-5MS coating, He carrier gas) and an Agilent 5975C mass spectrometer (ion source: EI+, 70 eV, 230 °C, quadrupole: 150 °C, interface: 300 °C) were used. The HPLC-MS measurements were performed with an Agilent Technologies 1200-type liquid chromatograph connected to a quadrupole mass analyzer and a combined APCI/ESI ion source, with which we were able to measure both in negative and positive mode. The eluents used were A—water, 5% acetonitrile, 0.05% TFA; B—acetonitrile, 5% water, and 0.075% TFA. The device also had a DAD 190–400 nm detector, with which we detected at 210 and 254 nm. The NMR spectroscopy tests were performed on a Bruker UltraShield Plus 500 and a Bruker UltraShield Plus 400 equipped with an automatic sample changer. CDCl_3_ and DMSO-d6 were used as solvents. Chemical shifts (δ) were given in ppm using solvent signals as internal standards, DMSO-d6 (1H δ 2.50 ppm, 13C δ 40.00 ppm). Coupling constants (J) were given in Hertz (Hz). The following were used to denote the splits: s (singlet), d (doublet), t (triplet), q (quartet), m (multiplet), brs (broad singlet), dd (doublet of doublet), td (doublet of triplet), qd (doublet of quartet), dt (triplet of doublet) (Supplementary Information S1; Figure S1).

The normal phase column chromatographic separations were performed using a Teledyne Isco CombiFlash Rf-type flash chromatographic device, using Teledyne Isco RediSep-type columns filled with normal-phase silica. The HR-MS measurements were performed with an Agilent Technologies 6230 TOF LC/MS mass analyzer with an ESI ion source connected to an Agilent Technologies 1200 liquid chromatograph or an Agilent 7890B GC with a Jeol AccuTof (JMS-T200GC) MS mass analyzer, and the samples were measured in either EI or FI mode. Elemental analytic measurements for 10a were carried out using a Thermo Fisher Flash EA 1112 Series CHNS-O Analyzer, with the oven temperature of 950 °C. The results of the measurements showed close matching with the expected atomic ratios (Table S1).

### 4.2. General Procedure for the Synthesis of 4-(S)-tBu Ligands and Complexes

Compounds **Lb** and **Lc** and silver and gold monocarbene complexes (**2b**–**f** and **3b**–**f**) were synthesized as described previously by Szabó et al. [24]. The synthesis of the copper complexes **1a**–**d**, **1f** is described in a manuscript under preparation [Szlávik et al.], while their general synthesis and characterization, including that of the respective ligands, are shown in Supplementary Information S2 and S3 and Figure S1.

Palladium complexes **4b**,**c** were synthesized through transmetallation from the corresponding silver complexes. The mixture of 170 mg **2b** or **2c** (0.32 mmol, 1.00 equivalent), allyl(chloro)palladium (32.1 mg, 0.55 equivalent, 0.18 mmol) in 1.6 mL dichloromethane (DCM) was stirred in an oven-dried vial for 1 h. The mixture was evaporated onto cellite and it was purified via normal-phase column chromatography using heptane and ethyl acetate as eluents. The NMR spectroscopic data are presented in Figure S1.

### 4.3. General Procedure for the Synthesis of SIPr Biscarbene Complexes ***9a*** and ***10a***

To obtain **9a**, to a Schlenk vial copper(I) tetrakis(acetonitrile) hexafluorophosphate 39.0 mg (1 equivalent, 0.10 mmol), NaO*t*Bu 26.1 mg (2.6 equivalent, 0.27 mmol) and 1,3-bis(2,6-diisopropyl-phenyl)-4,5-dihydroimidazol-1-ium tetrafluoroborate 100 mg (2 equivalent, 0.21 mmol) were added and the system was flushed with N_2_. Through a septum 2 mL anhydrous tetrahydrofuran was added and the mixture was stirred under inert conditions for 16 h. The vial was opened to air and the mixture was filtered, then pentane was added to the filtrate to form a precipitate. The mixture was filtered to obtain **9a** (57.2 mg, 0.06 mmol, yield: 58.8%) as a white solid, in accordance with a previous study [38]. NMR spectroscopic data are presented in Figure S1.

To obtain **10a**, a mixture of 100 mg **6a** (1 equivalent, 0.19 mmol) and AgBF_4_ (36.5 mg, 1 equivalent, 0.19 mmol) in 2 mL DCM was stirred at room temperature for 1 h in a brown vial. The mixture was filtered and the volatiles were evaporated. The mixture was left in air overnight, enabling the formation of AgO from the excess AgBF_4_, then dissolved in 1mL DCM and filtered again. The filtrate was added dropwise to 20 mL of pentane to form a precipitate. The precipitate was filtered to obtain **10a** (78.5 mg; 0.08 mmol; yield: 85.8%) as a white solid. NMR spectroscopic data and elemental analysis are presented in Figure S1.

### 4.4. Cell Lines and Culture Conditions

Mes-Sa and Mes-Sa/Dx5 cells were purchased from ATCC and kept in DMEM, supplemented with 10% fetal bovine serum, 5 mM glutamine, and 50 units/mL penicillin and streptomycin. Cells were tested and resulted as negative for mycoplasma contamination with the MycoAlert mycoplasma detection Kit (Lonza Group). For the cytotoxicity tests, we used the Mes-Sa uterine sarcoma cell line, and its MDR derivatives Mes-Sa/B1 and Mes-Sa/Dx5 were engineered to express fluorescent proteins [25]. CST cell line was established in our lab from a spontaneous tumor of a genetically engineered Brca1^−/−^, p53^−/−^ mouse strain [39], and was cultured in complete DMEM-F12. Cisplatin-resistant CST-2/3 was isolated after the in vivo cisplatin treatment of mice bearing CST tumors [Hámori et al., manuscript in preparation]. MSCL-1 mesenchymal cells were generated from human pluripotent stem cells and were cultured as described previously [26].

### 4.5. Cytotoxicity Tests

In a triple coculture system, cell lines expressing different fluorescent proteins were seeded in the same well. Cell lines (Mes-Sa mCherry, Mes-Sa/B1 mOrange, Mes-Sa/Dx5 eGFP [25]) were admixed and seeded on 384-well plates at a density of 3 × 800 cells/well (1:1:1 ratio), resulting in an overall total of 2400 cells. The next day, serially diluted compounds were added to the wells. Liquid handling was managed by a Hamilton StarLet pipetting robot. After 144 h of incubation, the fluorescence of mCherry (ex/em: 585/610 nm), mOrange (ex/em: 545/567 nm) and eGFP (ex/em: 485/510 nm) was recorded with an EnSpire plate reader (PerkinElmer, Waltham, MA, USA).

CST, a drug-naïve murine breast cancer cell line [39], and cisplatin-resistant cells CST-2/3 were seeded in monoculture into 384-well plates (Corning Inc., Corning, NY, USA) at a density of 2500 cells per well using automated liquid-handling. The cells were preincubated for 24 h to ensure adherence, followed by the addition of serially diluted compounds to a final well volume of 60 µL. Following 120 h of continuous drug exposure, cell viability was quantified using the PrestoBlue viability assay (Thermo Fisher Scientific, Waltham, MA, USA) after 1 h incubation at 555 nm excitation and 585 nm emission wavelengths.

The MSCL-1 line was seeded on 96-well plates with 15,000 cells/well density, and the following day, it was treated with the compounds for 72 h, when cell viability was assessed using a PrestoBlue viability assay.

pIC_50_ values derived from both co- and monoculture systems were determined by a custom program written in C# [25], then average and standard deviations were calculated and transformed to IC_50_ values. Every compound was tested in at least 3 independent experiments in triplicates.

### 4.6. H_2_DCFDA Test

Intracellular ROS production was assessed by the H_2_DCFDA—Cellular ROS Assay Kit (ab113851, Abcam, Cambridge, UK) according to the manufacturer’s instructions. Briefly, 20,000 cells (Mes-Sa and CST) were seeded on 96-well plates 1 day prior to the experiment. The cells were washed and incubated with H_2_DCFDA solution for 45 min in the dark. Following an additional washing step, test compounds were added in complete, phenol red free medium (FluoroBrite™, Thermo Fisher Scientific), at a final concentration of 25 µM. Fluorescence of 2′,7′-dichlorofluorescein (DCF) was measured at 484 nm/535 nm excitation/emission wavelengths. Data were normalized to baseline (0 h) values and represent at least three independent experiments.

### 4.7. Detection of Apoptosis

For cell death assessment, 150,000 cells were seeded in 24-well plates and treated the following day with the selected compounds for 72 h at concentrations corresponding to twice their respective IC_50_ values. Detached cells in the supernatant were collected, while adherent cells were trypsinized; the two suspensions were combined. After washing, cells were suspended in Annexin V Binding Buffer, followed by the addition of FITC-Conjugated Annexin V and propidium-iodide (Annexin V, FITC Apoptosis Detection Kit, Dojindo Laboratories, Kumamoto, Japan). Samples were incubated for 15 min at room temperature in the dark. Cell staining was analyzed using an Attune NxT flow cytometer, with quadrants set according to control measurements.

## 5. Conclusions

We characterized a series of N-heterocyclic carbene metal complexes with imidazolinium and imidazolium ligands and evaluated their cytotoxicity against both drug-sensitive and -resistant cancer cell lines. Our results demonstrate that the biological activity of these complexes is profoundly influenced by the nature of the metal center, ligand structure, and carbene multiplicity, with copper(I) and silver(I) complexes showing strong cytotoxicity, among which SIPr-based monocarbene complexes retained their activity even in clinically relevant in vitro resistance models. Altogether, our findings underline the therapeutic potential of 3-(2,6-diisopropyl-phenyl)-imidazolinium-based copper and silver NHC complexes, especially those without 4-(*S*)-*t*Bu substitution, as promising candidates for further development against resistant and refractory cancers. Future work will focus on in vivo validation and exploration of the precise molecular targets involved.

## Data Availability

The original contributions presented in this study are included in the article/Supplementary material. Further inquiries can be directed to the corresponding author.

## Supplementary Materials

The following supporting information can be downloaded at https://www.mdpi.com/article/10.3390/ijms262311382/s1.

## Abbreviations

The following abbreviations are used in this manuscript:

NHC	N-heterocyclic carbene.
MDR	Multidrug resistance.
4-(*S*)-*t*Bu	4-(*S*)-*tert*-butyl
RR	Resistance ratio (IC_50___MDR_/IC_50___parental_).
ROS	Reactive oxygen species.
GC-MS	Gas Chromatography–Mass Spectrometry.
HPLC	High-Performance Liquid Chromatography.
NMR	Nuclear Magnetic Resonance.
DCM	Dichloromethane.
TFA	Trifluoroacetic acid.
DMEM	Dulbecco’s Modified Eagle Medium
