# Supplementary material for: Imidazolinium-Based NHC–Metal Complexes Overcome Both Cancer Multidrug Resistance and Cisplatin Resistance In Vitro
Source: Int J Mol Sci. 2025 Nov 25;26(23):11382. doi: 10.3390/ijms262311382 (PMC12692346; doi:10.3390/ijms262311382)
Supplement: Supplementary file 1 [file ijms-26-11382-s001.zip › ijms-3950001-supplementary.pdf]

SUPPORTING INFORMATION for

Imidazolinium-Based NHC–Metal Complexes Overcome Both Cancer Multidrug Resistance and Cisplatin Resistance In Vitro

Márton Szlávik [1,2,†], Lidia Ines Haffarases [3,4,†], Réka Mandel [3], Fanni Fekecs [1], Ágota Apáti [3], Attila Paczal [1], András Kotschy [1], Gergely Szakács [3,5] and Szilárd Tóth [3,6,*]

[1] Servier Research Institute of Medicinal Chemistry, Záhony Utca 7, H-1031 Budapest, Hungary;

marton.szlavik.part@servier.com (M.S.); fekecsfanni99@gmail.com (F.F.); attila.paczal@servier.com (A.P.); andras.kotschy@servier.com (A.K.)

[2] Hevesy György PhD School of Chemistry, Eötvös Loránd University, Pázmány Péter Sétány 1/A, H-1117 Budapest, Hungary

[3] Institute of Molecular Life Sciences, HUN-REN Research Centre for Natural Sciences, Magyar Tudósok Körútja 2, H-1117 Budapest, Hungary; haffaressas.ines.lidia@ttk.hu (L.I.H.); rekamandel@gmail.com (R.M.); apati.agota@ttk.hu (Á.A.); szakacs.gergely@ttk.hu (G.S.)

[4] Doctoral School of Biology, Institute of Biology, Eötvös Loránd University, Pázmány Péter Sétány 1/C, H-1117 Budapest, Hungary

[5] Center for Cancer Research, Medical University of Vienna, Spitalgasse 23, A-1090 Vienna,

[6] Austria National Laboratory for Drug Research and Development, H-1117 Budapest, Hungary

[*] Correspondence: toth.szilard.enzim@ttk.hu; Tel.: +36-(1)-382-6737

[†] These authors contributed equally to this work.

Table of content

Supplementary Information S1. Characterization of synthetized compounds **4b**, **4c**, **9a** and **10a**

Supplementary Information S2. General synthesis and characterization of the 4-(*S*)-*t*Bu ligands

Supplementary Information S3. General synthesis and characterization of the copper complexes **1a-d**, **f**.

Supplementary Figure S1. ^{13}C, ^{1}H and ^{19}F NMR spectra of the synthesized compounds

Supplementary Table S1. Elemental analysis of **10a**

Supplementary Table S2. IC_{50} values against the Mes-Sa uterine sarcoma cell lines

Supplementary Table S3. IC_{50} values against the CST murine breast cancer cell lines

Supplementary Table S4. IC_{50} values against the MSCL-1 mesenchymal stem cell like cell line

Supplementary Figure S2. Structure of **1b*** and **1c***

Supplementary Information S1.

Characterization of synthetized compounds **4b**, **4c**, **9a** and **10a**

Allyl-[(4S)-4-tert-butyl-3-(2,6-diisopropylphenyl)-1-[(1S)-1-phenylethyl]imidazolidin-2-ylidene]-chloro-palladium (**4b**). Yield: 159.2 mg (0.28 mmol, 87%) white solid.

^{1}H NMR (400 MHz, CDCl3) δ ppm 7.83/7.74 (d/d, *J* = 7.4/*J* = 7.4 Hz, 2 H), 7.46-7.37 (m/m, 2 H), 7.32-7.29 (m/m, 1 H), 7.28 (t, 1 H), 7.27-7.19 (m/m, 1 H), 7.15-7.11 (m/m, 1 H), 6.71/6.37 (q/q, *J* = 6.9/*J* = 7.0 Hz, 1 H), 5.26-5.12/4.71-4.60 (m/m, 2 H), 4.17/4.15/3.19/3.01 (d+d/d+d, *J* = 7.7 Hz, *J* = 7.7Hz/ *J* = 13.5, *J* = 13.0 Hz, 2 H), 4.20-4.10/3.89-3.80 (m/m, 1 H), 3.98/3.96 (dd/dd, *J* = 12.1, 5.6 Hz/*J*= 12.1, 5.5 Hz, 1 H), 3.70/3.65/2.98/2.92 (t+dd/t+dd, *J* = 11.8 , *J* = 21.9, 11.6 Hz/*J* = 5.7, *J* = 11.2, 5.6 Hz, 2 H), 3.09/2.57/2.1/1.34 (d+d/d+d, *J* = 13.2, *J* = 6.7 Hz/ *J* = 11.7, *J* = 5.3 Hz, 3 H), 2.85-2.76 (m, 1 H), 1.70/1.58 (d/d, *J* = 7.1Hz/*J* = 7.0 Hz, 3 H), 1.65/1.51/1.34/1.26 (d+d/d+d, *J* = 6.6, *J* = 6,5 Hz/ *J* = 7.1, *J* = 6.7 Hz, 6 H), 1.37/1.36/1.32 (d+d+d, *J* = 6.7 Hz/*J* = 6.6 Hz/*J* = 6.1 Hz, 6 H), 0.68 (s, 9 H). ^{13}C NMR (100 MHz, CDCl3) δ ppm 212.7/211.2, 148.0, 144.4, 139.0, 138.6/138.5, 128.4/128.4, 128.3/128.2, 128.0/128.0, 127.8/127.8, 125.9, 124.5/124.4, 115.5/114.5, 74.0/73.9, 73.8/73.6, 56.9/56.6, 47.4/46.8, 46.0/45.7, 35.8, 29.3, 28.7/28.7, 28.5, 28.3/28.2, 26, 16.4/16.1. HRMS (EI) M+($C_{30}H_{43}ClN_2$).: calcd. for: 572.2150, found = 572.2185 (δ = 7.1 ppm).

Allyl-[(4S)-4-tert-butyl-3-(2,6-diisopropylphenyl)-1-[(1R)-1-phenylethyl]imidazolidin-2-ylidene]-chloro-palladium (**4c**). Yield: 154.1 mg (0.27 mmol, 67.5%) white solid.

^{1}H NMR (400 MHz, CDCl3) δ ppm 7.44-7.30 (m, 6 H), 7.28-7.22 (m, 1 H), 7.16-7.10 (m, 1 H), , 6.59/6.28 (q/q, *J* = 6.9/*J* = 6.9 Hz, 1 H), 5.11-5.0/4.70-4.56 (m/m, 1 H), 4.13/4.11/3.10/2.98 (d+d/d+d, *J* = 10.4, 10.4/*J* = 13.2, 13.2 Hz, 2 H), 4.12-4.08/3.85-3.76 (m/m, 1 H), 3.97 (p, *J* = 5.8 Hz, 1 H), 3.54/3.50/3.43/3.34 (dd+t/dd+t, *J* = 11.5, 6.9, *J* = 11.6/*J* = 11.3, 6.5, *J* =11.6 Hz, 2 H), 3.07/2.48/1.94/1.30 (d+d/d+d, *J* = 6.7, *J* = 5.9/*J* = 11.6, *J* = 8,6 Hz, 3 H), 2.82-2.68 (m, 1 H), 1.90/1.80 (d/d, *J* = 7.0/*J* = 7.0 Hz, 3 H), 1.63/1.49/1.29/1.27 (d+d/d+d, *J*= 6.6, *J* = 6.5/ *J* = 6.6, *J* =6.5 Hz, 6 H), 1.35-1.27 (m, 6 H), 0.87/0.86 (s/s, 9 H). ^{13}C NMR (100 MHz, CDCl3) δ ppm 213.3/211.7, 148.1/147.9, 144.7/144.6, 140.5/140.3, 139.5/139.2, 128.6/128.6, 128.1/128.1, 127.6, 126.8/126.7, 125.8, 124.4/124.4, 115.7/114.4, 74.3/74.1, 73.5/73.3, 57.9/57.5, 48.3/47.2, 45.7/45.4, 35.8/35.8, 28.9, 28.7/28.6, 28.2/28.1, 26.0/25.9, 24.7/24.7, 24.6/24.3, 16.9/16.5. HRMS (EI) M+($C_{30}H_{43}ClN_2$).: calcd. for: 572.2150, found = 572.2285 (δ = 24.6 ppm).

bis[1,3-bis(2,6-diisopropylphenyl)imidazolidin-2-ylidene]copper(1+);tetrafluoroborate (**9a**). Yield: 57.2 mg (0.06 mmol, 58.8%) white solid.

^{1}H NMR (400/500 MHz, CDCl3) δ ppm 7.43 (t, *J* = 7.7 Hz, 4 H), 7.24 (d, *J* = 7.7 Hz, 8 H), 4.08 (s, 8 H), 3.00 (sp, *J* = 6.7 Hz, 8 H), 1.34/1.23 (d+d, *J* = 6,6, *J* = 6.6 Hz, 48 H).^{13}C NMR (400/500

MHz, CDCl3) δ ppm 200.9, 146.5, 133.9, 130.1, 124.7, 53.9, 28.8, 25.5/23.9. ^{19}F NMR (376 MHz, CDCl3) δ ppm -153.2.

bis[1,3-bis(2,6-diisopropylphenyl)imidazolidin-2-ylidene]silver(1+);tetrafluoroborate (**10a**). Yield: 78.5 mg (0.08 mmol, 85.8%) white solid.

^{1}H NMR (400 MHz, CDCl3) δ ppm 7.37 (t, *J* = 7.8 Hz, 4 H), 7.07 (d, *J* = 7.8 Hz, 8 H), 3.87 (s, 8 H), 2.76 (sp, *J* = 6.9 Hz, 8 H), 1.2/0.77 (d+d, *J* = 6,9, *J* = 6.9 Hz, 48 H) ^{13}C NMR (100 MHz, CDCl3) δ ppm 205.3, 146.2, 134.7, 130, 124.8, 54.4, 28.5, 25.4/24. ^{19}F NMR (376 MHz, CDCl3) δ ppm -154.7

<u>**Supplementary Information S2.**</u>

General synthesis of the 4-(*S*)-*t*Bu-imidazolinium ligands **La-f**. Synthesis of **La** is described here, while synthesis of **Lb-f** was described in Szabó et al (Organometallics, 2020. 39(19): p. 3572-3589).

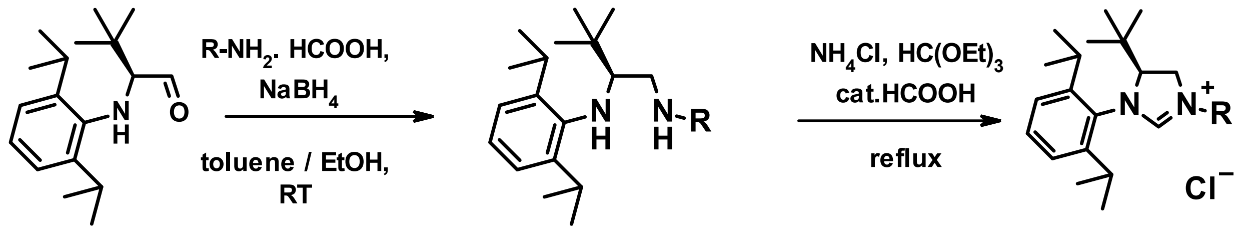

L a-f

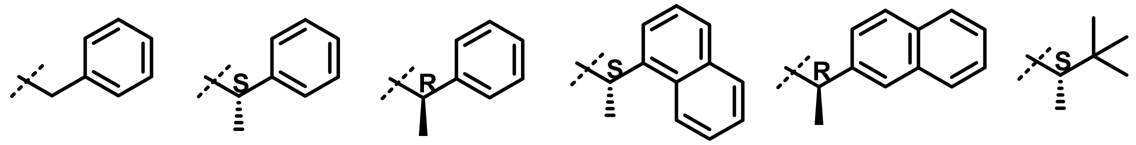

a b c d e f

Synthesis and characterization of **(2*S*)-N1-benzyl-N2-(2,6-diisopropylphenyl)-3,3-dimethyl-butane-1,2-diamine**

(2S)-2-(2,6-diisopropylanilino)-3,3-dimethyl-butanal (1 equiv.) was measured into a round bottom flask equipped with a PTFE coated magnetic stirring bar and 5 mL/mmol Toluene and catalytic amount of HCOOH was added. To the mixture 1 equivalent of the respective amine was measured, and the mixture was stirred at room temperature until the reaction reached full conversion. The solvent was evaporated under reduced pressure, then the mixture was

dissolved in EtOH. 2 equivalent sodium borohydride was added in one batch and the mixture was stirred at room temperature until it reached full conversion. The reaction mixture was then quenched with the addition of 2 mL/mmol of (2M) ammonium chloride solution and extracted with ethyl acetate. The organic layer was collected and dried over anhydrous $MgSO_4$, then the crude product was purified via flash chromatography on silica gel using heptane and ethyl acetate. Synthesis was started from 1880 mg (6.83 mmol) (2S)-2-(2,6-diisopropylanilino)-3,3-dimethyl-butanal. Yield: 1369 mg (3.73 mmol 55%) yellowish oil.

1H NMR (400 MHz, DMSO-D6) δ ppm 7.21 (m, 2 H), 7.15 (m, 1 H), 7.05 (dm, 2 H), 7 (d, J = 7.6 Hz, 2 H), 6.89 (dd, J = 8.1, 7.6 Hz, 1 H), 3.49/3.45 (d+d, J = 13.7 Hz, J = 13.7 Hz, 2 H), 3.36 (m, 2 H), 3.02 (m, 1 H), 2.64/2.53 (dd+dd, J = 12.3, 3.4 Hz, J = 12.4, 5.8 Hz, 2 H), 1.15/1.12 (d+d, J = 6.8 Hz, J = 6.1 Hz, 12 H), 1 (s, 9 H). 13C NMR (100MHz, DMSO-D6) δ ppm 143.0, 141.2,140.6,128.4, 127.9, 126.8, 123.9, 122.3, 67.2, 53.7, 50.7, 35.8, 27.7, 27.4, 24.5, 24.4.

HRMS [M + H]+ calculated C25H38N2 = 367.3108, found = 367.3111 (δ = 0.9 ppm).

Synthesis and characterization of **(4*S*)-1-benzyl-4-tert-butyl-3-(2,6-diisopropylphenyl)-4,5-dihydroimidazol-1-ium chloride**

The respective diamine was measured into a round bottom flask and 5 mL/mmol of triethyl orthoformate added. To the reaction mixture subsequently 1.2 equivalent ammonium chloride and 0.1 equivalent formic acid was measured. The flask was mounted with a condenser and the mixture was heated up to 130°C and stirred on the given temperature until it reached full conversion. After the reaction reached full conversion, the mixture was let to cool down to room temperature, and it was purified via normal phase chromatography using dichloromethane.

(4*S*)-1-benzyl-4-tert-butyl-3-(2,6-diisopropylphenyl)-4,5-dihydroimidazol-1-ium chloride was synthesized starting from 1570 mg (4.28 mmol) 2n. Yield: 1360 mg (3.29 mmol 77%) white solid.

1H NMR (500 MHz, DMSO-D6) δ ppm 9.27 (s, 1 H), 7.52-4.73 (m, 6 H), 7.38 (dd, J = 7.9, 1.5 Hz, 1 H), 7.36 (dd, J = 7.9, 1.5 Hz, 1 H), 4.98/4.78 (d+d, J = 14.7 Hz, J = 14.7 Hz, 2 H), 4.41 (dd, J = 12.2, 9.1 Hz, 1 H), 4.15/3.94 (t+dd, J = 12.3 Hz, J = 12.5, 8.9 Hz, 2 H), 3.28-6.22 (m, 1 H), 2.93-2.83 (m, 1 H), 1.30 (dd, J = 6.5, 1.8 Hz, 6 H), 1.23/1.13 (d+d, J = 6.7 Hz, J = 6.7 Hz 6 H), 0.74 (s, 9 H). 13C NMR (125MHz, DMSO-D6) δ ppm 160.6, 146.0, 145.8, 134.0, 132.4, 130.8, 129.5, 129.3, 129.0, 125.9, 125.7, 74.8, 51.6, 50.1, 35.4, 28.8, 28.4, 26.6, 26.0, 25.8, 23.6, 23.0.

HRMS (ESI) [M]+ calculate C26H37N2 = 377.2952; found = 377.2957 (δ = 0.2 ppm).

Supplementary Information S3.

General synthesis and characterization of the copper complexes **1a-d**, **f**.

The respective imidazolinium salt, 2 equivalent of K_2CO_3 and 1 equivalent of CuCl were measured into a Schlenk vial equipped with a magnetic stirring bar, then the vial was inerted with alternated vacuum and N_2 gas and sealed with a septum. 5 mL/mmol acetone was added through the septum and the mixture was heated up to 60°C and stirred until the reaction reached full conversion (1 hour - 4 hours). After the reaction reached full conversion, the mixture was let to cool down to room temperature and celite was added to the mixture, then it was purified via normal phase chromatography using DCM and MeOH as eluents.

Synthesis and characterization of **[(4S)-1-benzyl-4-tert-butyl-3-(2,6-diisopropylphenyl) imidazolidin-2-yl]-chloro-copper (1a)**

1a was synthesized stating from 200 mg (0.48 mmol) **La.** Yield: 111 mg (0.23 mmol 48%), white solid.

^{1}H NMR (500 MHz, $CDCl_3$) δ ppm 7.46-7.33 (m, 6 H), 7.23 (dd, *J* = 7.5, 1.3 Hz, 1 H), 7.17 (dd, *J* = 7.7, 1.3 Hz, 1 H), 5.01/4.80 (d+d, *J* = 14.7 Hz, J= 14.7 Hz, 2 H), 4.01 (dd, *J* = 11.8, 10.3 Hz, 1 H), 3.63/3.42 (t+t, *J* = 11.7 Hz, *J* = 10.7 Hz, 2 H), 3.56-3.46 (m, 1 H), 2.80-2.71 (m, 1 H), 1.54/1.34 (d+d, *J* = 6.6 Hz, *J* = 6.6 Hz, 6 H), 1.33/1.27 (d+d, *J* = 6.6 Hz, *J* = 6.6 Hz, 6 H), 0.75 (s, 9 H). ^{13}C NMR (125MHz, $CDCl_3$) δ ppm 204.5, 146.6, 146.0, 136.5, 135.0, 129.2, 129.1, 128.5, 127.9, 125.2, 125.0, 76.2, 55.2, 50.2, 34.8, 29.0, 28.2, 27.4, 26.1, 23.5, 23.2.

HRMS (ESI) [M-Cl+CH3CN]+ calculated $C_{26}H_{36}ClCuN_2$, found = 480.2438 (δ = 0.7 ppm).

Synthesis and characterization of **[(4S)-4-tert-butyl-3-(2,6-diisopropylphenyl)-1-[(1S)-1-phenylethyl]imidazolidin-2-yl]-chloro-copper (1b)**

1b was synthesized from 1000 mg (2.34 mmol) **Lb**. Yield: 788 mg (1.61 mmol 69%).

^{1}H NMR (500 MHz, DMSO-d6) δ ppm 7.48-7.37 (m, 5 H), 7.36 (t, *J* = 7.4 Hz, 1 H), 7.27 (dd, *J* = 13.2, 1.6 Hz, 1 H), 7.25 (dd, *J* = 13.0, 1.6 Hz, 1 H), 5.42 (q, *J* = 7.1 Hz, 1 H), 3.90 (dd, *J* = 12.0, 10.0 Hz, 1 H), 3.65/3.52 (dd+t, *J* = 12.0, 9.8 Hz, *J* = 12.0 Hz, 2 H), 3.48-3.42 (m, 1 H), 2.71-2.64 (m, 1 H), 1.75 (d, *J* = 7.1 Hz, 3 H), 1.39 (d, *J* = 6.8 Hz, 3 H), 1.29 (d, *J* = 6.8 Hz, 3 H), 1.26 (d, *J* = 6.8 Hz, 3 H), 1.11 (d, *J* = 6.7 Hz, 3 H), 0.73 (s, 9 H). ^{13}C NMR (125 MHz, DMSO-d6) δ ppm 202.6, 146.2, 146.1, 140.2, 137.5, 129.2, 125.4, 125.2, 75.0, 58.5, 48.0, 34.7, 28.7, 28.1, 27.2, 26.4, 26.2, 23.6, 23.4, 19.1.

HRMS (EI) [M]+ calculated $C_{27}H_{38}ClCuN_2$488.2020, found = 488.2106 (δ = 18.8 ppm).

Synthesis and characterization of **[(4S)-4-tert-butyl-3-(2,6-diisopropylphenyl)-1-[(1R)-1-phenylethyl]imidazolidin-2-yl]-chloro-copper (1c)**

1c was synthesized from 150 mg (0.35 mmol) **Lc.** Yield: 115 mg (0.23 mmol 67%), white solid.

^{1}H NMR (500 MHz, DMSO-d6) δ ppm 7.49-7.33 (m, 5 H), 7.39-7.37 (m, 1 H), 7.29-7.26 (m, 1 H), 7.26-7.23 (m, 1 H), 5.32 (q, *J* = 7.0 Hz, 1 H), 4.01-3.91 (m, 1 H), 4.01-3.91/3.27 (m+m, 2 H), 3.43-3.34 (m, 1 H), 2.86-2.74 (m, 1 H), 1.78 (d, *J* = 7.1 Hz, 3 H), 1.38/1.26 (d+d, *J* = 6.8 Hz, *J* = 6.8 Hz, 6 H), 1.32/1.15 (d+d, *J* = 6.9 Hz, *J* = 6.7 Hz, 6 H), 0.63 (s, 9 H). ^{13}C NMR (125 MHz, DMSO-d6) δ ppm 202.8, 146.5, 146.5,140.0,137.6, 129.4, 129.2, 128.5, 127.0, 125.4, 125.2) 128.5, 125.4, 125.2, 75.5, 58.5, 47.3, 28.8, 28.1, 27.3/23.3, 26.4, 26.1/23.4, 18.3.

HRMS (EI) [M]+ calculated $C_{27}H_{38}ClCuN_2$488.2020, found = 488.2136 (δ = 25.0 ppm).

Synthesis and characterization of **[(4S)-4-tert-butyl-3-(2,6-diisopropylphenyl)-1-[(1S)-1-(1-naphthyl)ethyl]imidazolidin-2-yl]-chloro-copper (1d)**

1d was synthesized stating from 862 mg (1.81 mmol) **Ld.** Yield: 790 mg (1.46 mmol 81%), white solid.

^{1}H NMR (500 MHz, $CDCl_3$) δ ppm 8.36 (d, *J* = 7.9 Hz, 1 H), 7.91 (d, *J* = 8.8 Hz, 1 H), 7.89 (d, *J* = 10.5 Hz, 1 H), 7.62-7.50 (m, 4 H), 7.32 (t, *J* = 7.7 Hz, 1 H), 7.19 (dd, *J* = 7.7, 1.5 Hz, 1 H), 7.14 (dd, *J* = 7.7, 1.5 Hz, 1 H), 6.23 (q, *J* = 6.9 Hz, 1 H), 3.89 (dd, *J* = 12.0, 9.9 Hz, 1 H), 3.60/2.71 (t+dd, *J* = 11.7 Hz, *J* = 11.5, 10.0 Hz, 2 H), 3.48-3.40 (m, 1 H), 2.79-2.71 (m, 1 H), 1.97 (d, *J* = 6.9 Hz, 3 H), 1.61/1.25 (d+d, *J* = 6.8 Hz, *J* = 6.8 Hz, 6 H), 1.32/1.22 (d+d, *J* = 6.9 Hz, *J* = 6.8 Hz, 6 H), 0.40 (s, 9 H). ^{13}C NMR (125 MHz, $CDCl_3$) δ ppm 204.4, 146.6, 146.0, 134.0, 133.1, 131.7, 129.6, 129.2, 129.0, 127.1, 126.3, 125.1, 125.0, 125.0, 124.5, 123.5, 75.3, 55.1, 46.2, 28.6, 28.2, 27.4/23.3, 26.4, 26.2/23.5, 18.9.

HRMS (ESI) [M-Cl+CH3CN]+ calculated $C_{31}H_{40}ClCuN_2$, found = 544.2751 (δ = 0.6 ppm).

Synthesis and characterization of **[(4S)-4-tert-butyl-3-(2,6-diisopropylphenyl)-1-[(1S)-1,2,2-trimethylpropyl]imidazolidin-2-yl]-chloro-copper (1f)**

1f was synthesized stating from 200 mg (0.49 mmol) **Lf**. Yield: 141 mg (0.30 mmol 61%) white solid.

^{1}H NMR (500 MHz, $CDCl_3$) δ ppm 7.33 (t, *J* = 7.6 Hz, 1 H), 7.19 (dd, *J* = 7.8, 1.5 Hz, 1 H), 7.14 (dd, *J* = 7.7, 1.5 Hz, 1 H), 4.27 (q, *J* = 7.3 Hz, 1 H), 4.03 (dd, *J* = 11.8, 10.3 Hz, 1 H), 3.85/3.60 (t+t, *J* = 11.6, *J* = 10.8 Hz, 2 H), 3.45 (sp, *J* = 6.9 Hz, 1 H), 2.72 (sp, *J* = 6.8 Hz, 1 H), 1.48 (d, *J* = 6.8 Hz, 3 H), 1.37 (d, *J* = 7.1 Hz, 3 H), 1.35 (d, *J* = 7.1 Hz, 3 H), 1.32 (d, *J* = 7.1 Hz, 3 H), 1.22 (d, *J* = 6.7 Hz, 3 H), 1.08 (s, 9 H), 0.82 (s, 9 H). ^{13}C NMR (125 MHz, $CDCl_3$) δ ppm 205.9, 146.7, 145.9, 136.9, 129.1, 125.0, 125.0, 75.7, 65.1, 48.5, 35.5, 34.7, 29.0, 28.2, 27.9, 27.1, 26.7, 26.0, 23.5, 23.2, 14.0.

HRMS (ESI) [M-Cl+CH3CN]+ calculated $C_{25}H_{42}ClCuN_2$, found = 474.2908 (δ = 0.8 ppm).

Supplementary Figure S1. ^{13}C, ^{1}H and ^{19}F NMR spectra of synthesized compounds

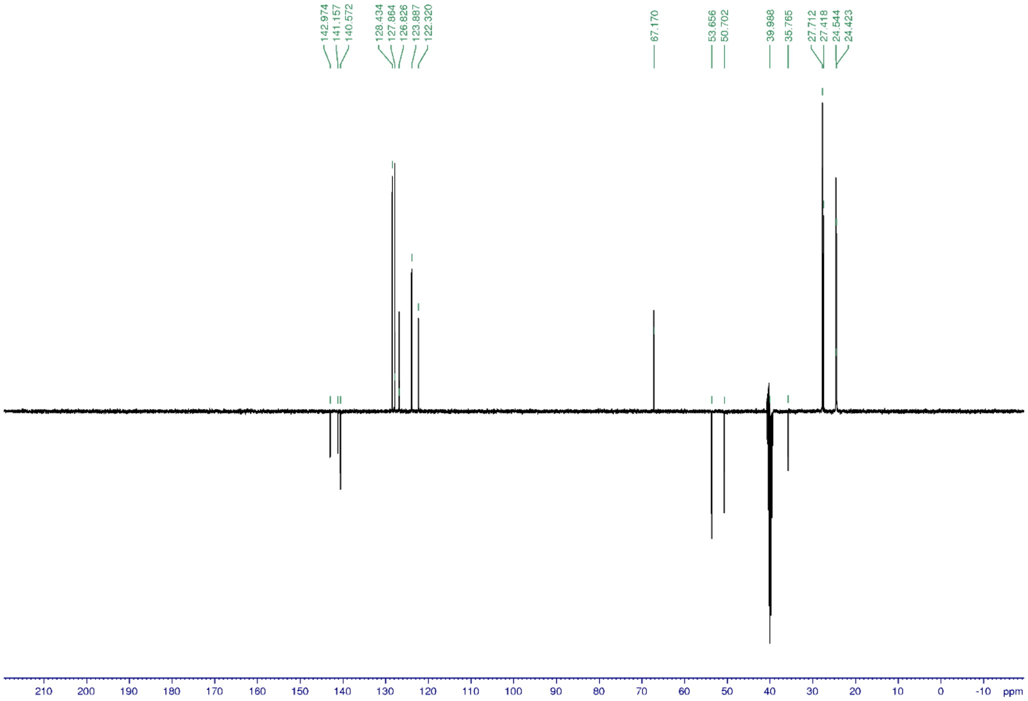

^{13}C NMR (100 MHz, dmso-d6) spectra of **(2*S*)-N1-benzyl-N2-(2,6-diisopropylphenyl)-3,3-dimethyl-butane-1,2-diamine**

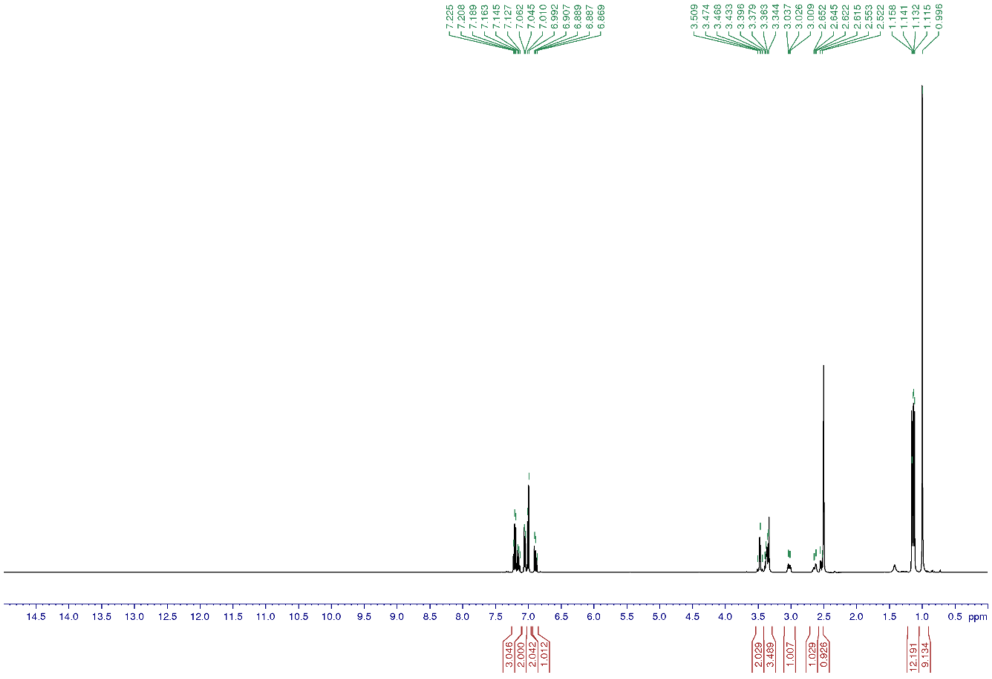

^{1}H NMR (400 MHz, dmso-d6) spectra of **(2*S*)-N1-benzyl-N2-(2,6-diisopropylphenyl)-3,3-dimethyl-butane-1,2-diamine**

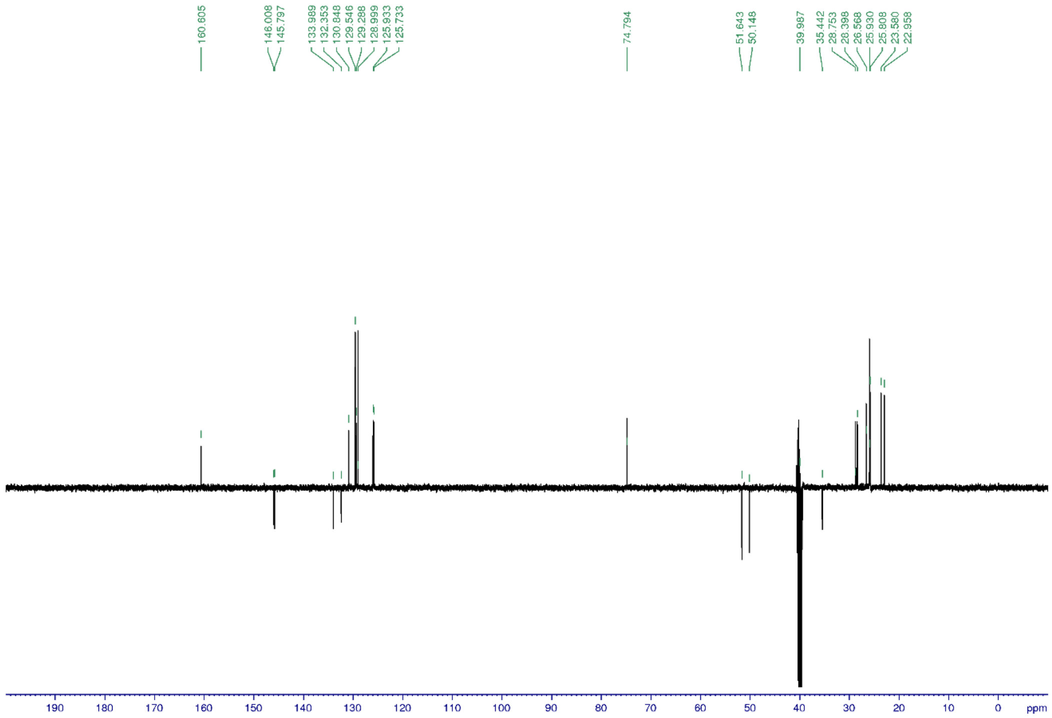

^{13}C NMR (100 MHz, dmso-d6) spectra of **(4*S*)-1-benzyl-4-tert-butyl-3-(2,6-diisopropylphenyl)-4,5-dihydroimidazol-1-ium chloride**

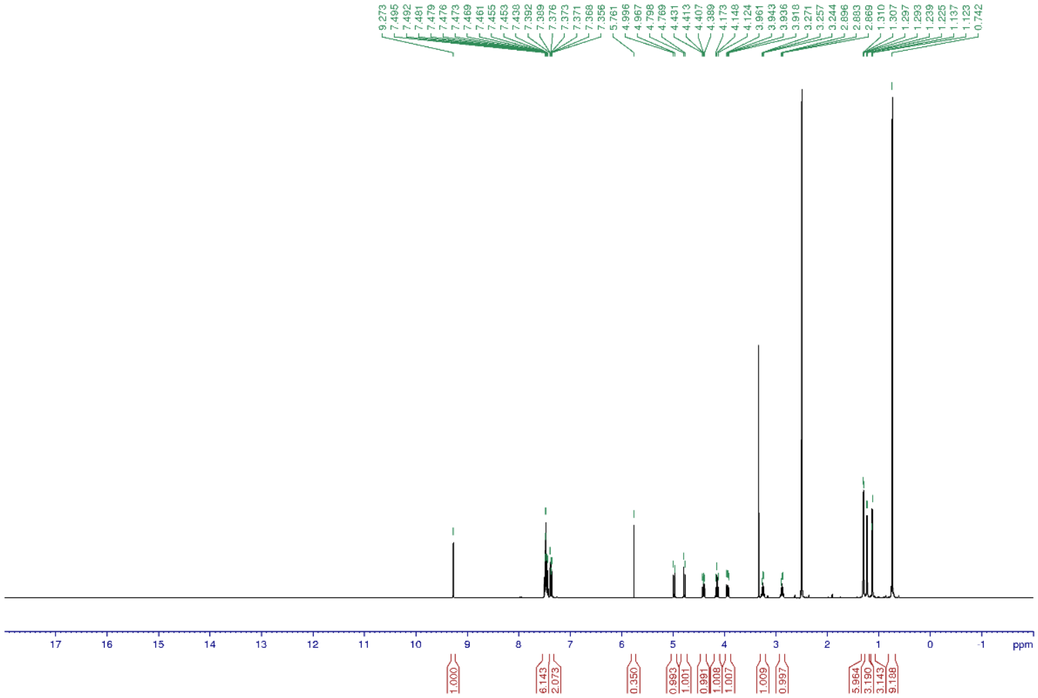

^{1}H NMR (400 MHz, dmso-d6) spectra of **(4*S*)-1-benzyl-4-tert-butyl-3-(2,6-diisopropylphenyl)-4,5-dihydroimidazol-1-ium chloride**

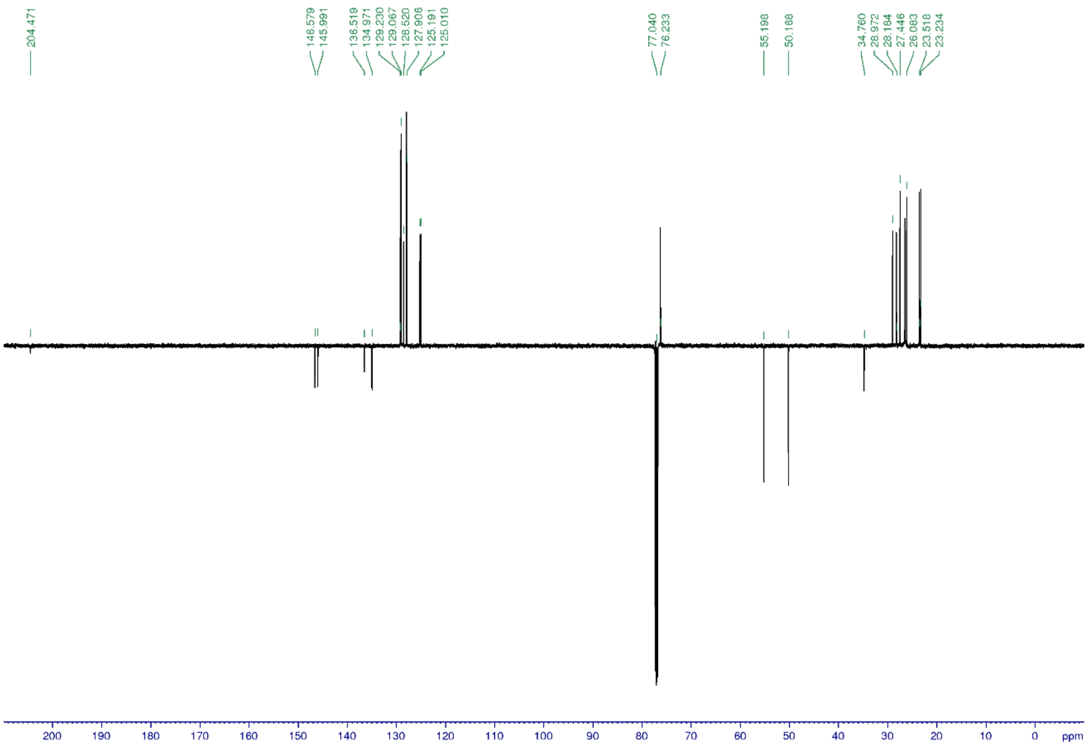

^{13}C NMR (100 MHz, dmso-d6) spectra of **1a**

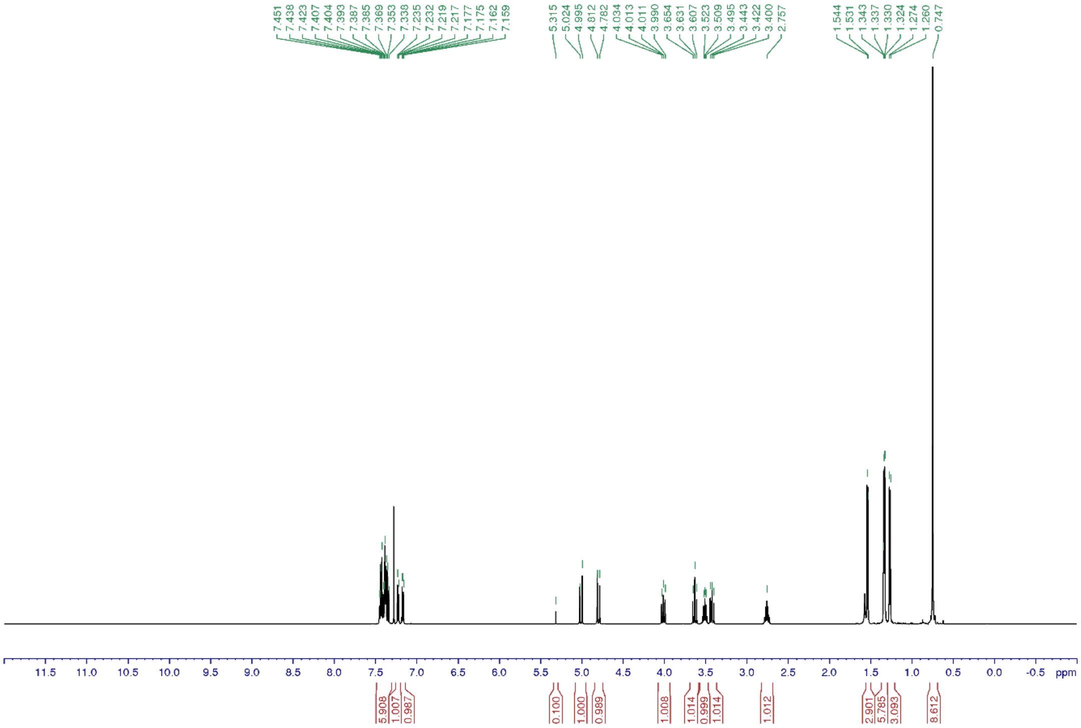

^{1}H NMR (400 MHz, dmso-d6) spectra of **1a**

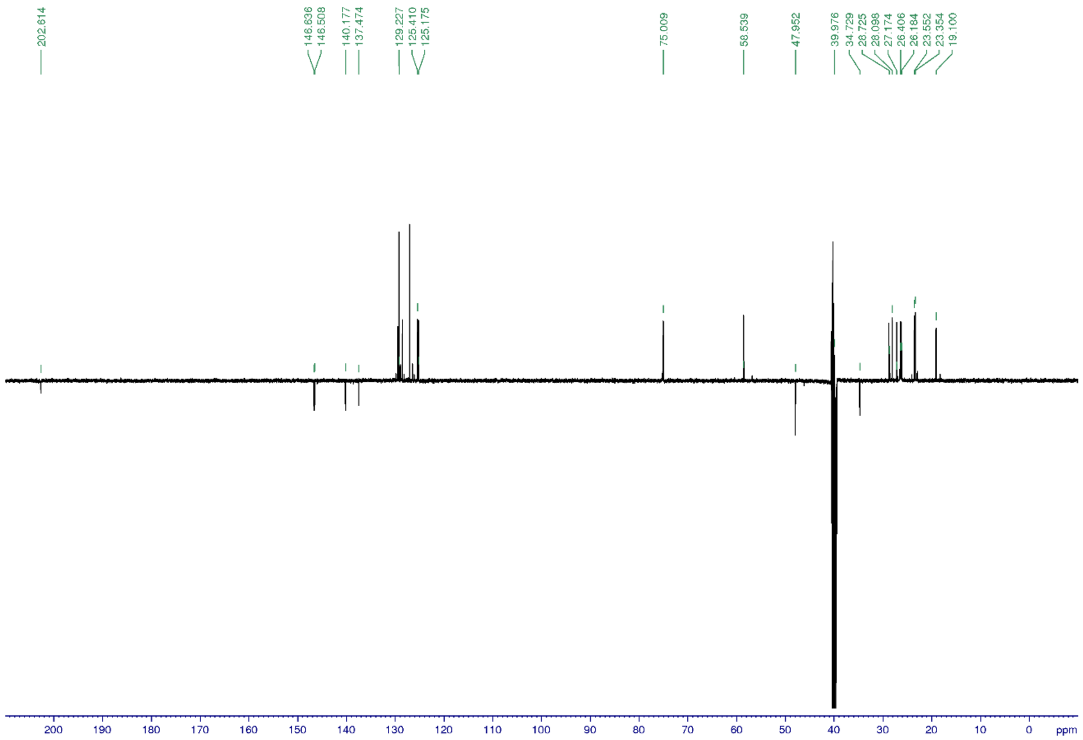

^{13}C NMR (100 MHz, dmso-d6) spectra of **1b**

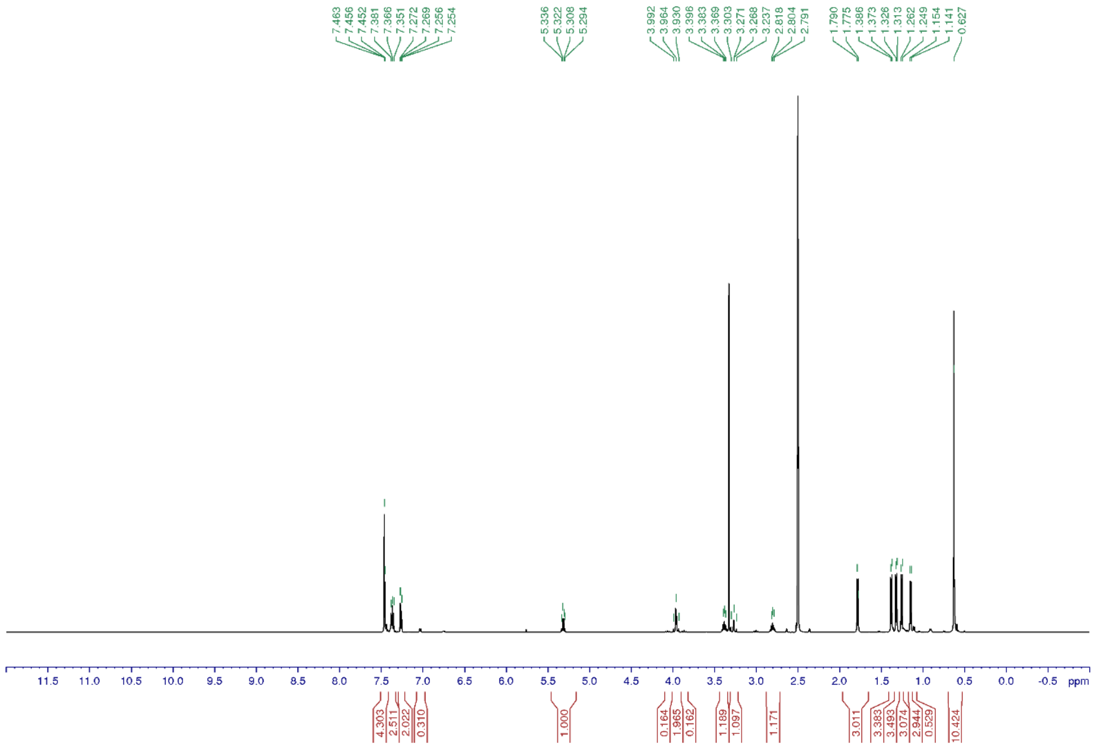

^{1}H NMR (400 MHz, dmso-d6) spectra of **1b**

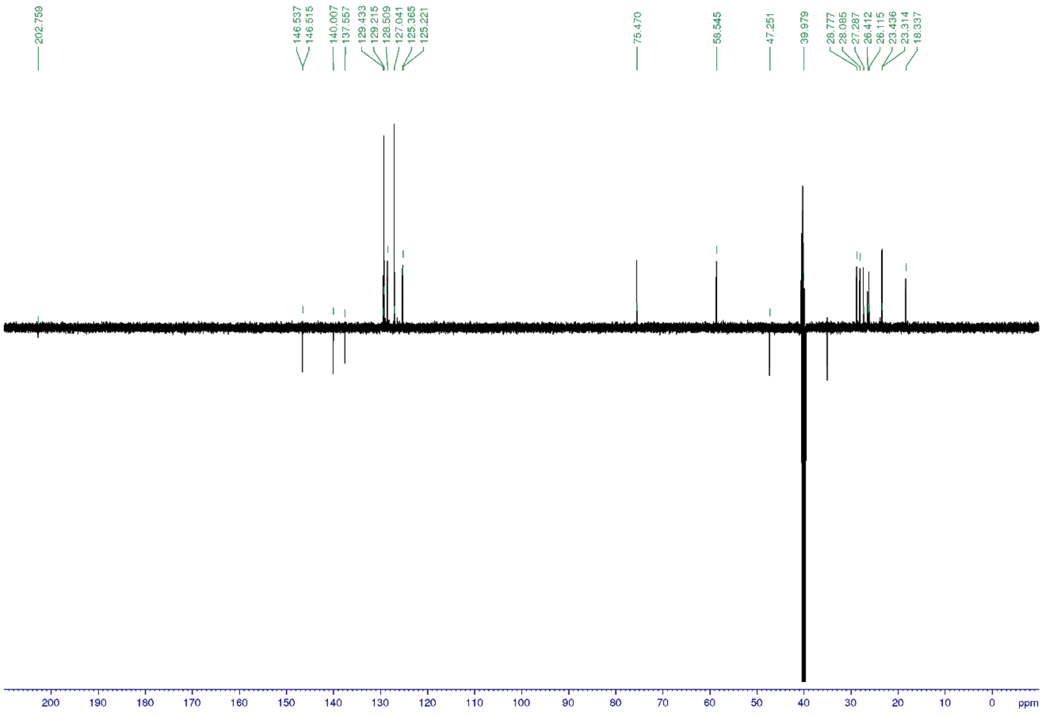

^{13}C NMR (100 MHz, dmso-d6) spectra of **1c**

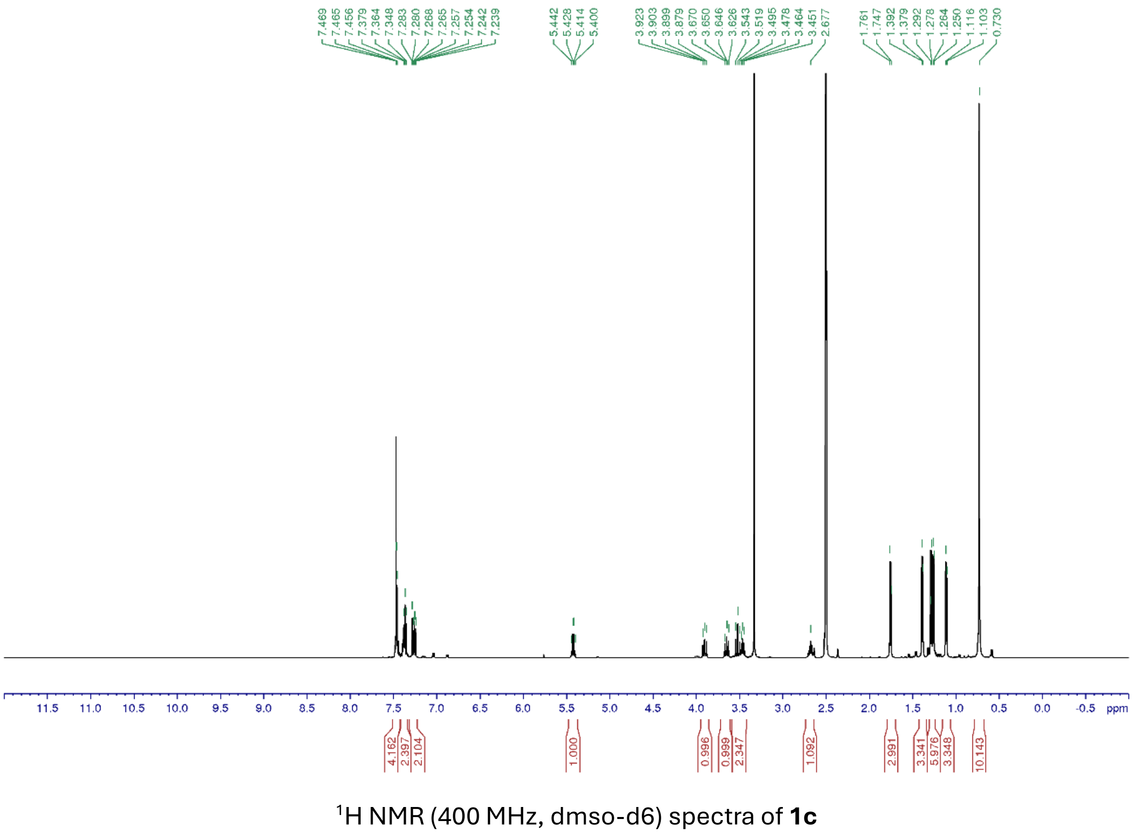

^{1}H NMR (400 MHz, dmso-d6) spectra of **1c**

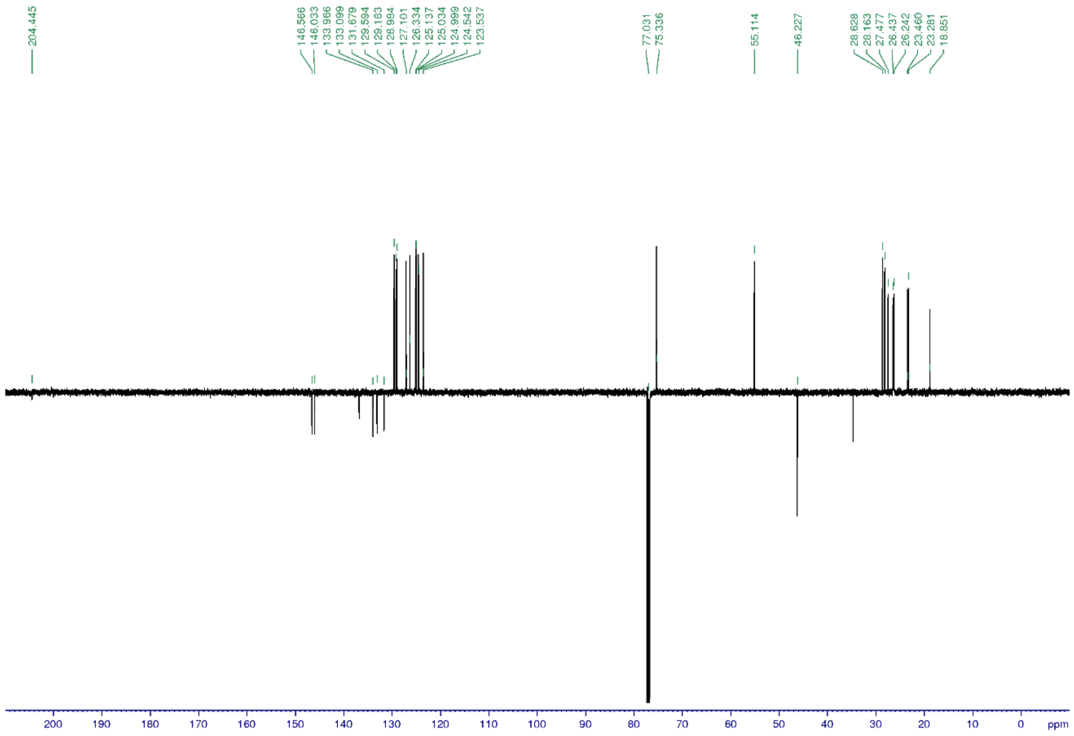

^{13}C NMR (100 MHz, dmso-d6) spectra of **1d**

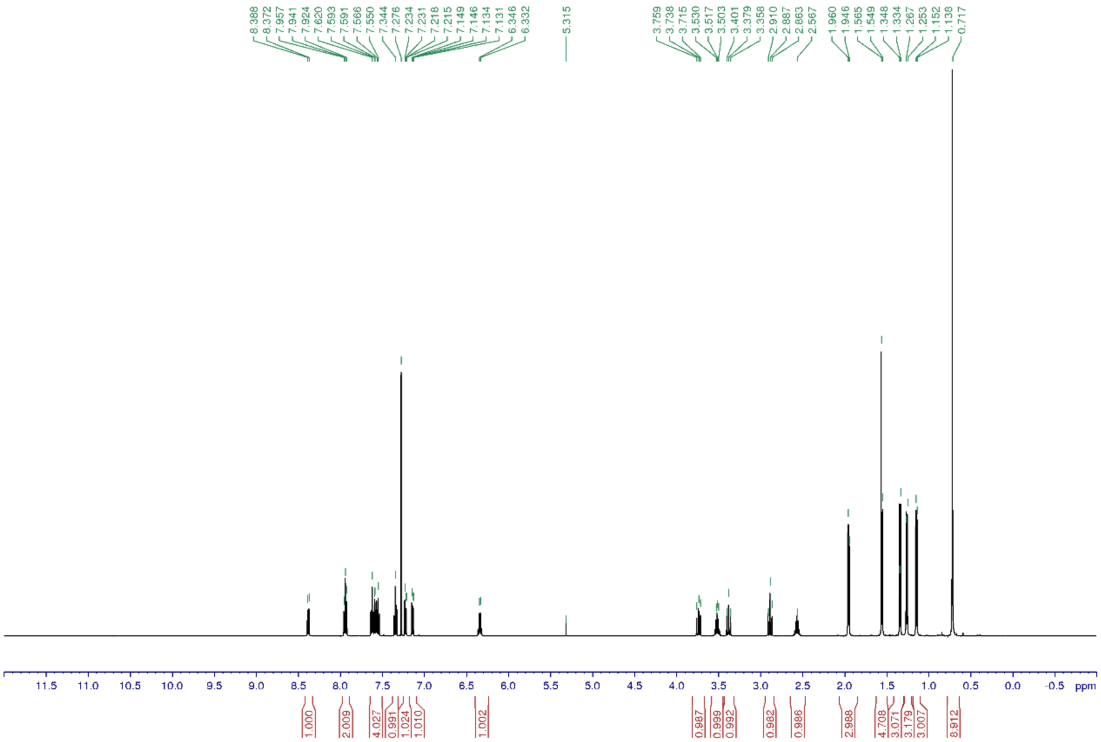

^{1}H NMR (400 MHz, dmso-d6) spectra of **1d**

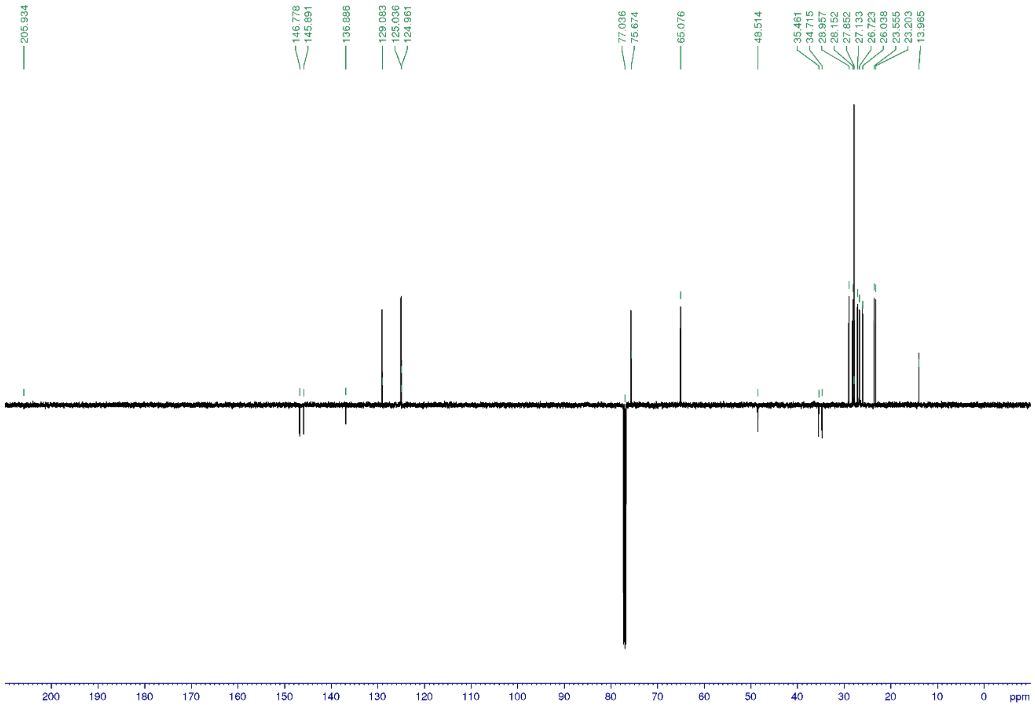

^{13}C NMR (100 MHz, dmso-d6) spectra of **1f**

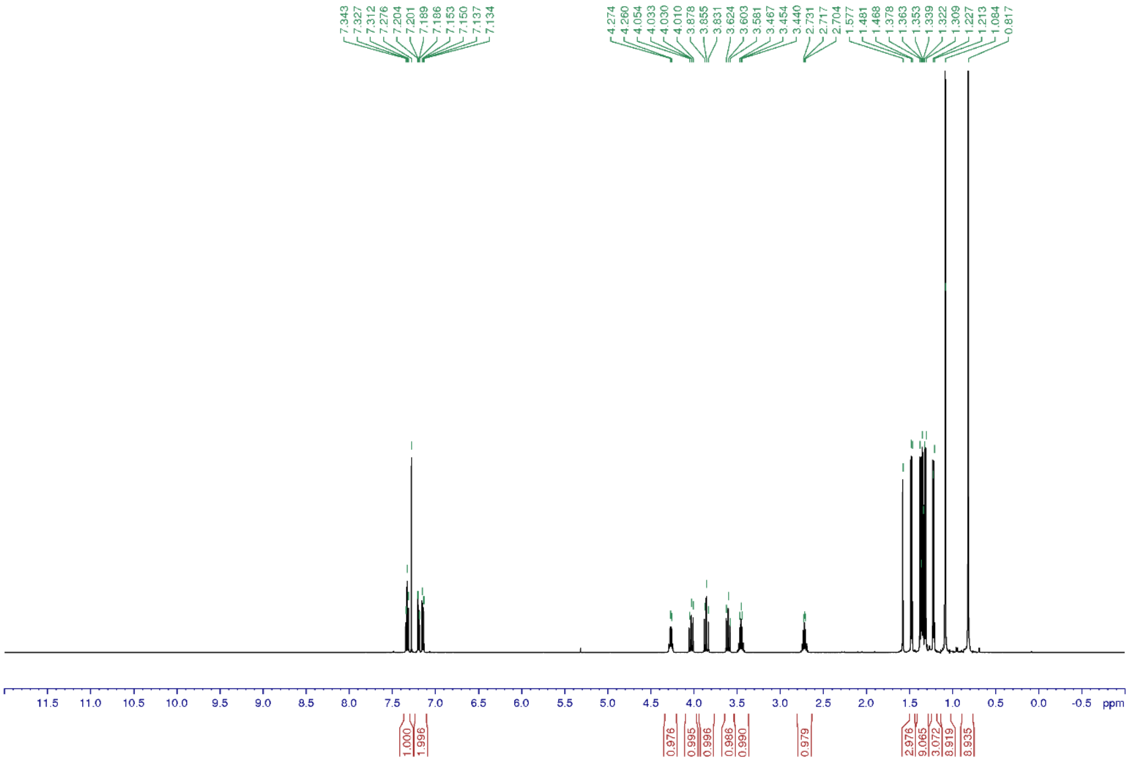

^{1}H NMR (400 MHz, dmso-d6) spectra of **1f**

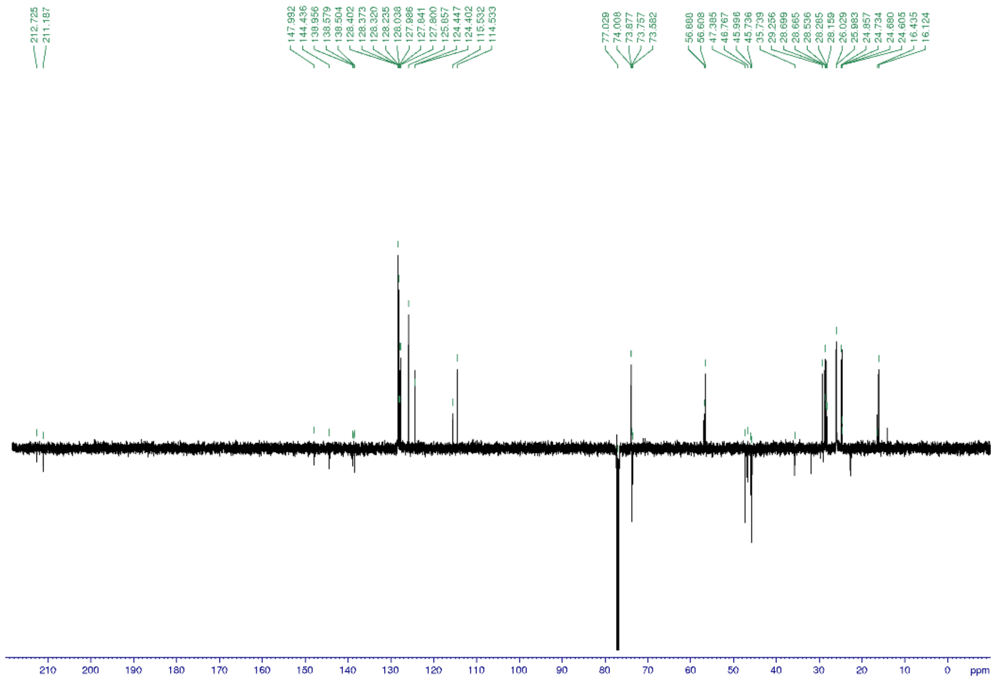

^{13}C NMR (100 MHz, dmso-d6) spectra of **4b**

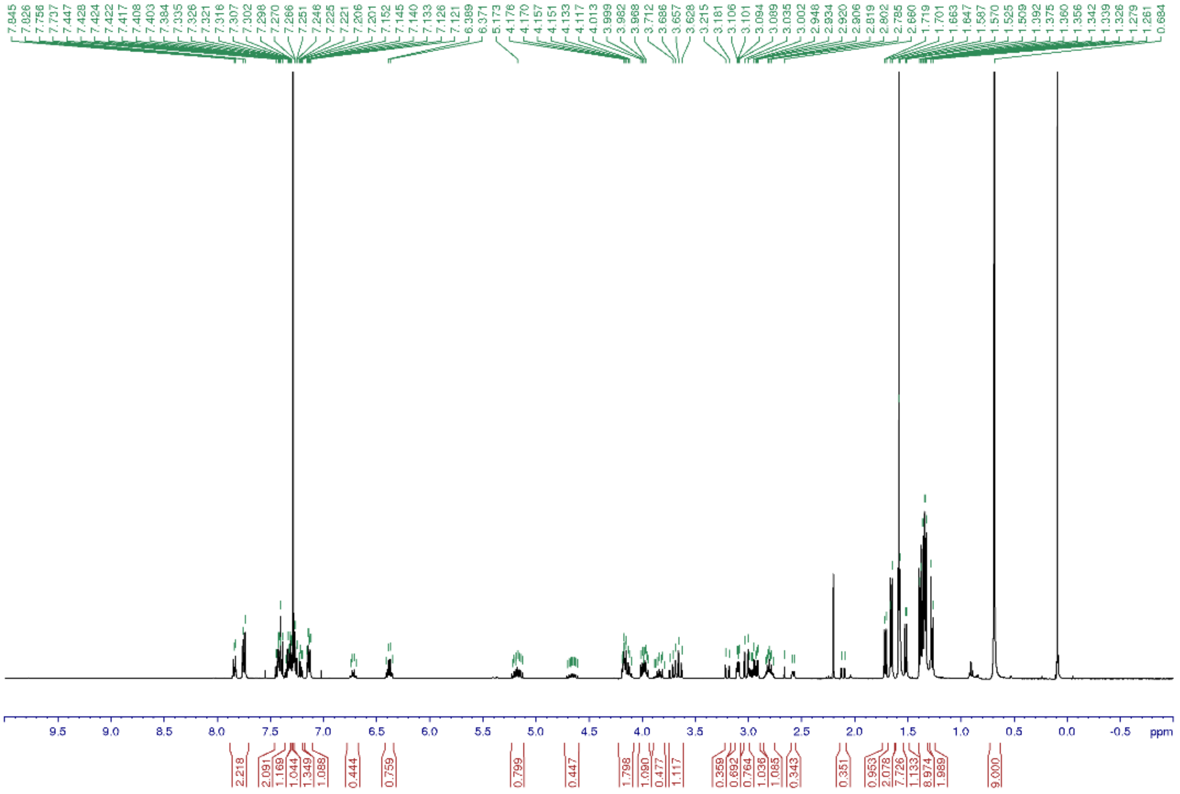

^{1}H NMR (400 MHz, dmso-d6) spectra of **4b**

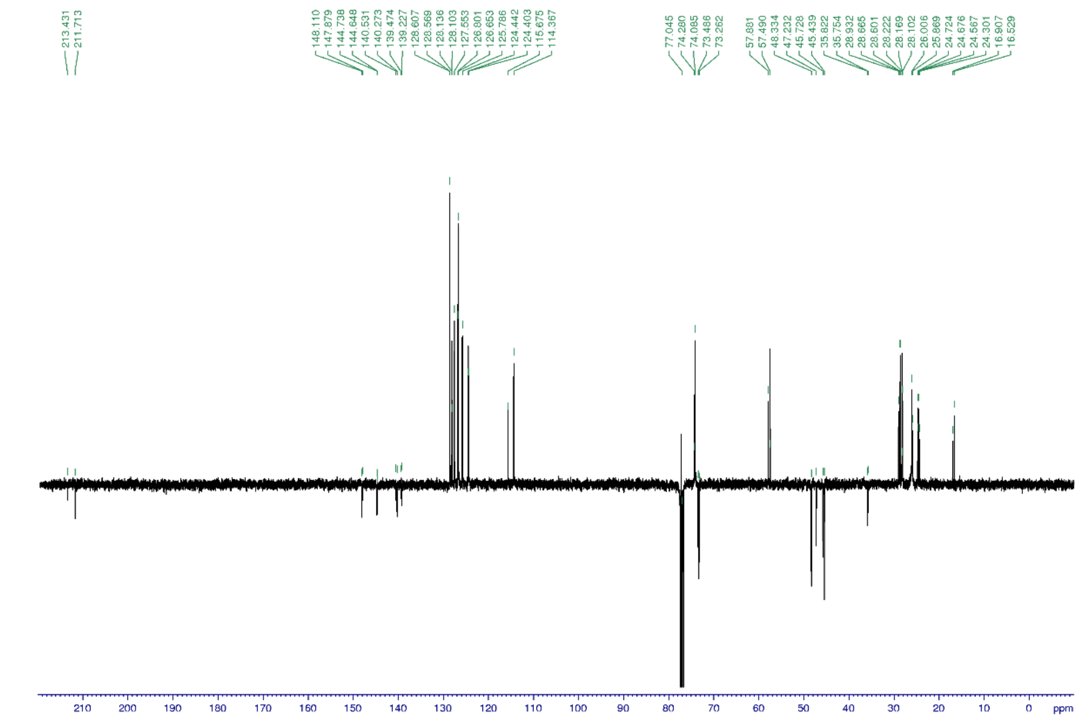

^{13}C NMR (100 MHz, dmso-d6) spectra of **4c**

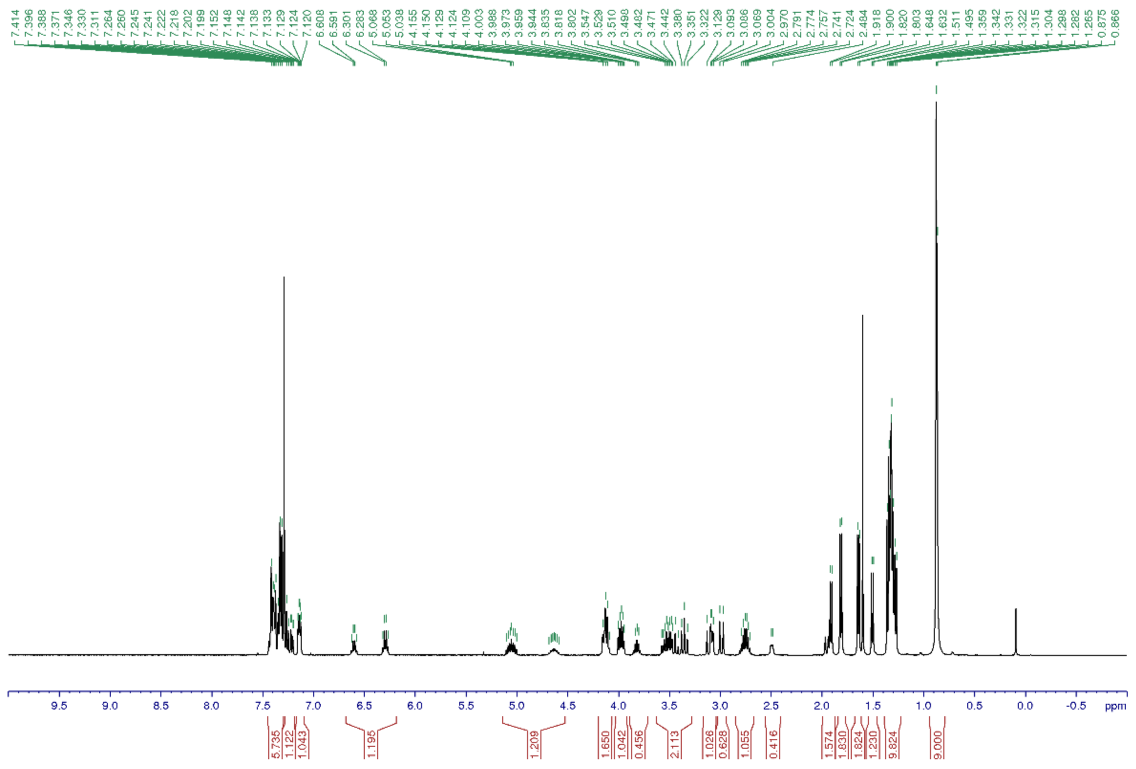

^{1}H NMR (400 MHz, dmso-d6) spectra of **4c**

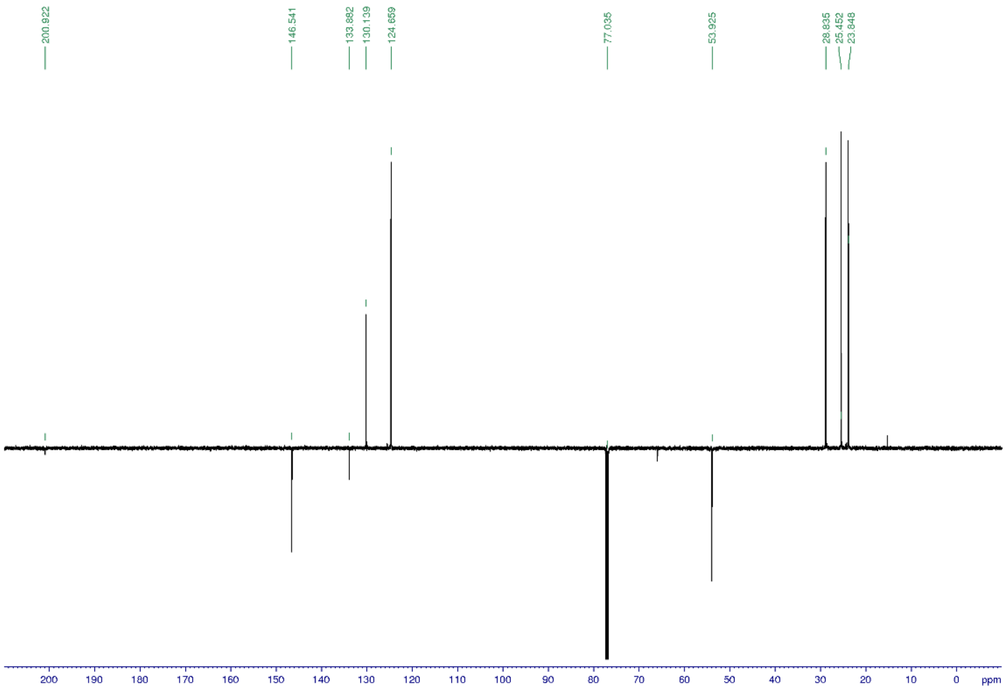

^{13}C NMR (100 MHz, dmso-d6) spectra of **9a**

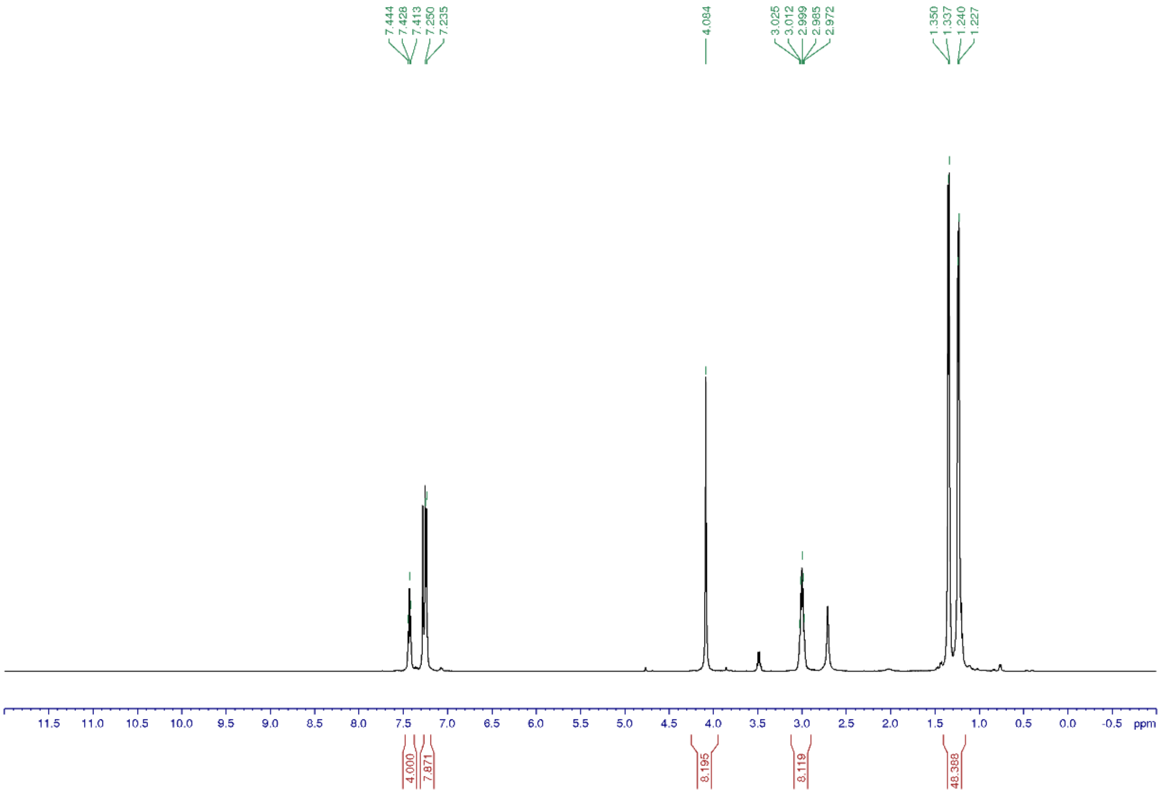

^{1}H NMR (400 MHz, dmso-d6) spectra of **9a**

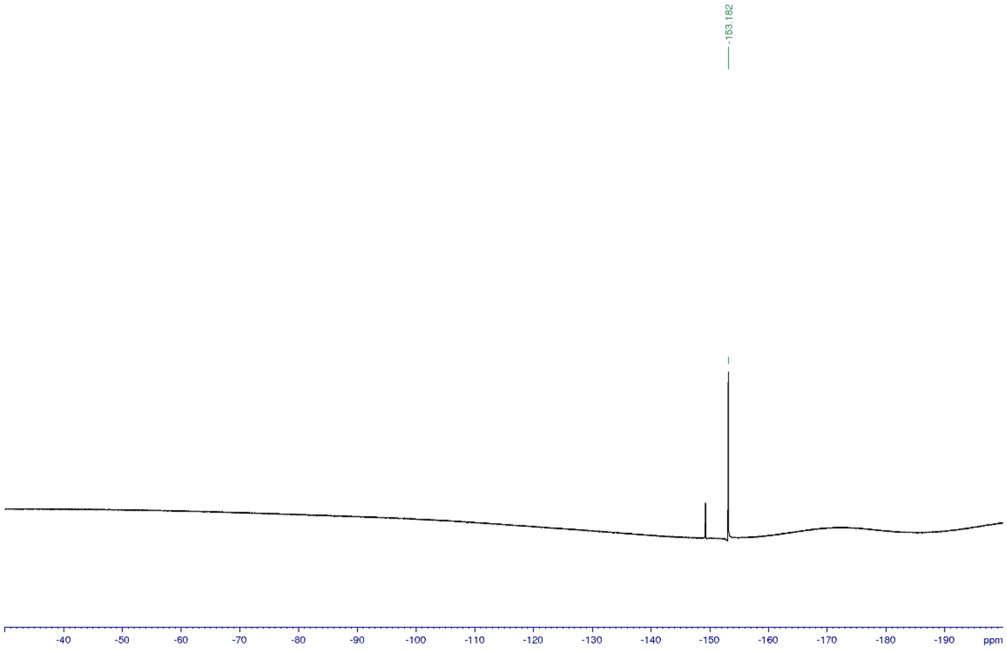

^{19}F NMR (376 MHz, dmso-d6) spectra of **9a**

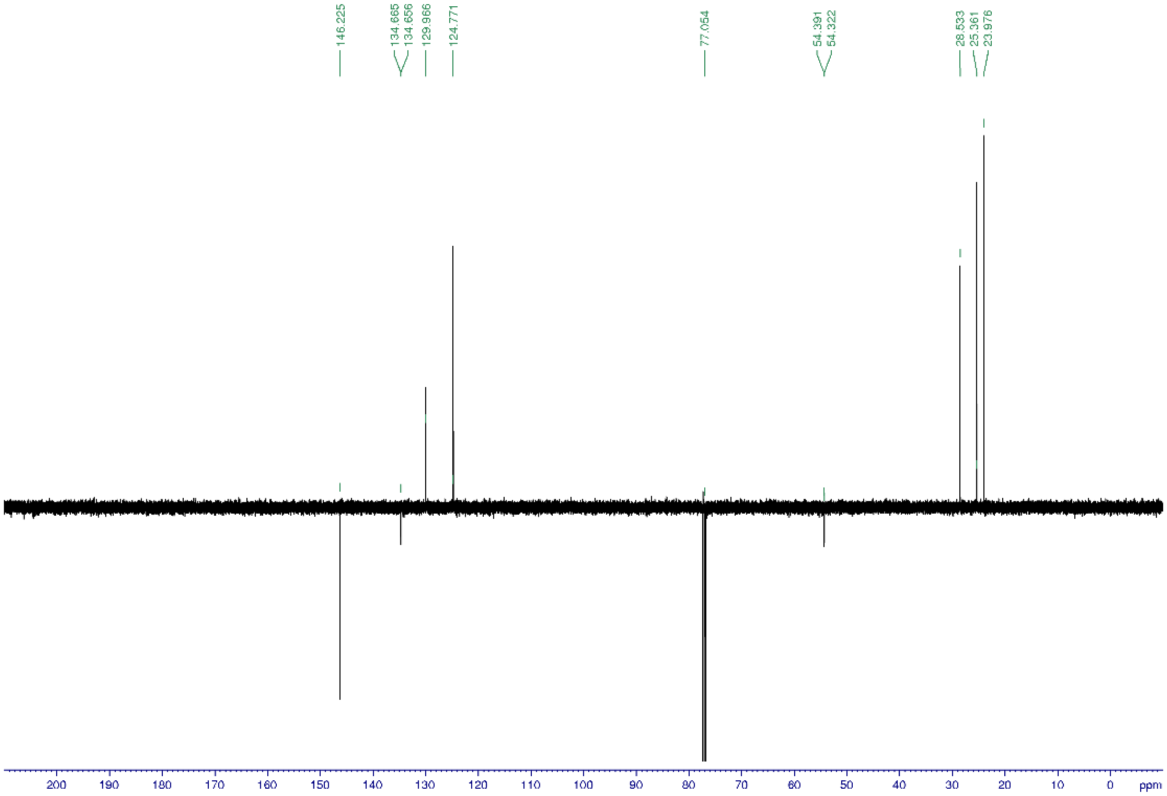

^{13}C NMR (100 MHz, dmso-d6) spectra of **10a**

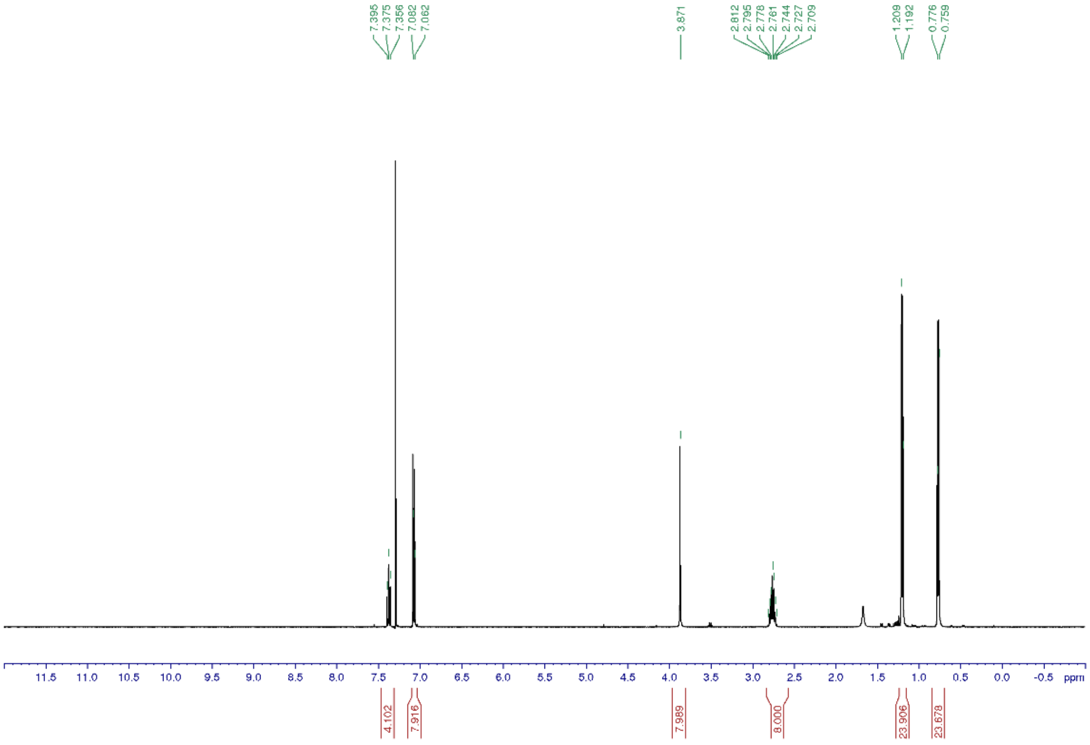

^{1}H NMR (400 MHz, dmso-d6) spectra of **10a**

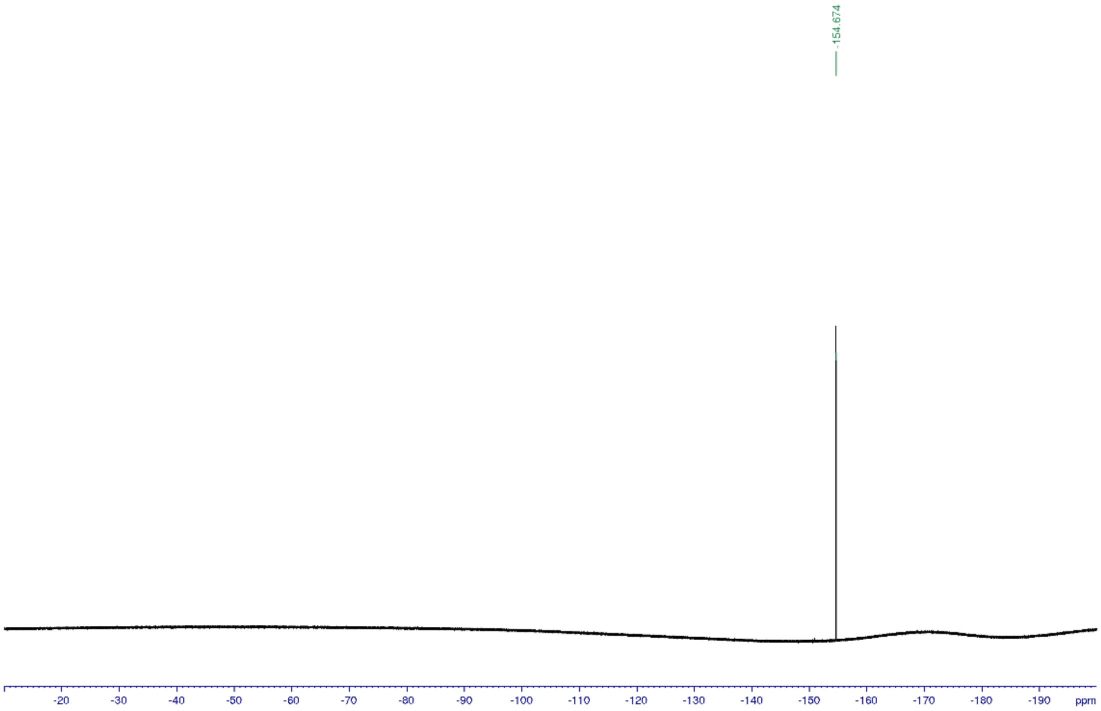

^{19}F NMR (376 MHz, dmso-d6) spectra of **10a**

Supplementary Table S1. Elemental analysis of **10a**

<table>
<tr><th colspan="3">Molecular Data</th><th>Structure</th></tr>
<tr><td>Type</td><td>Principal</td><td>Principal</td><td rowspan="5">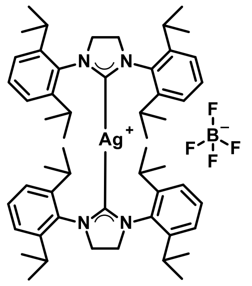
</td></tr>
<tr><td>Theoretical</td><td>1</td><td>1</td></tr>
<tr><td>Molecular Weight</td><td>889.08</td><td>86.80</td></tr>
<tr><td>Exact Mass</td><td>889,5272</td><td>87,0035</td></tr>
<tr><td>Formula</td><td>C54 H76 Ag N4</td><td>B F4</td></tr>
</table>

Element	Theory	Results		Mean	Deviation
**C**	66.46	63.96	64.27	64.12	-2.26
**H**	7.85	7.51	7.44	7.48	-0.38
**N**	5.74	5.36	5.33	5.35	-0.39
**Ag**	11.05				
**B**	1.11				
**F**	7.79				

Supplementary Table S2. IC_{50} values with the standard deviation (SD) of the tested compounds against the fluorescent uterine sarcoma cell lines Mes-Sa, Mes-Sa/B1 and Mes-Sa/Dx5. RR: resistance ratio ($IC_{50\_MDR\ line}$ / $IC_{50\_parental\ line}$) of Mes-Sa/B1 (RR_B1) and of Mes-Sa/Dx5 (RR_Dx5).

Compound	Metal center	Mes-Sa mCherry		Mes-Sa/B1 mOrange		Mes-Sa/Dx5 eGFP		RR_B1	RR_Dx5
		IC_{50} [µM]	+SD/-SD	IC_{50} [µM]	+SD/-SD	IC_{50} [µM]	+SD/-SD		
**1a**	Cu(I)	0.52	*0.12/0.10*	2.45	*0.44/0.38*	0.69	*0.07/0.06*	4.7	1.3
**1b**		1.74	*0.44/0.35*	8.51	*0.65/0.60*	2.32	*0.81/0.60*	4.9	1.3
**1b***		0.75	*0.07/0.07*	5.25	*2.10/1.50*	1.46	*0.15/0.13*	7.0	1.9
**1c**		0.80	*0.12/0.10*	5.50	*1.99/1.46*	1.18	*0.16/0.14*	6.9	1.5
**1c***		0.74	*0.19/0.15*	3.97	*1.82/1.25*	2.18	*1.26/0.80*	5.4	2.9
**1d**		0.51	*0.08/0.07*	4.94	*2.44/1.63*	1.74	*0.35/0.29*	9.8	3.4
**1f**		3.04	*0.94/0.72*	8.13	*0.89/0.80*	4.19	*1.26/0.97*	2.7	1.4
**2b**	Ag(I)	0.75	*0.13/0.11*	14.34	*1.10/1.02*	5.50	*0.70/0.62*	19.1	7.3
**2c**		1.69	*0.40/0.32*	14.39	*3.11/2.56*	7.14	*0.73/0.66*	8.5	4.2
**2d**		0.54	*0.20/0.15*	5.20	*1.44/1.13*	3.37	*1.86/1.20*	9.6	6.2
**2e**		0.93	*0.23/0.19*	7.37	*0.79/0.71*	5.25	*1.83/1.36*	7.9	5.6
**2f**		0.74	*0.07/0.07*	3.77	*0.43/0.39*	1.37	*0.39/0.30*	5.1	1.8
**3b**	Au(I)	6.17	*0.59/0.54*	14.79	*5.86/4.20*	9.92	*3.84/2.77*	2.4	1.6
**3c**		18.62	*3.26/2.77*	39.81	*12.67/9.61*	39.58	*10.13/8.07*	2.1	2.1
**3d**		7.81	*1.47/1.24*	21.88	*3.53/3.04*	19.44	*3.72/3.12*	2.8	2.5
**3e**		7.47	*1.05/0.92*	19.05	*2.23/1.99*	16.85	*2.28/2.00*	2.6	2.3
**3f**		11.39	*3.22/2.51*	18.62	*3.61/3.03*	13.80	*5.21/3.78*	1.6	1.2
**4b**	Pd(II)	5.19	*0.84/0.72*	9.33	*3.64/2.62*	12.23	*4.58/3.33*	1.8	2.4
**4c**		2.34	*0.16/0.15*	5.62	*1.31/1.06*	10.06	*3.05/2.34*	2.4	4.3
**5a**	Cu(I)	0.42	*0.07/0.06*	0.44	*0.07/0.06*	0.43	*0.13/0.10*	1.0	1.0
**5b**		1.01	*0.22/0.18*	2.00	*0.66/0.50*	1.83	*0.89/0.60*	2.0	1.8
**6a**	Ag(I)	0.61	*0.16/0.13*	0.98	*0.28/0.22*	0.89	*0.32/0.23*	1.6	1.5
**6b**		1.82	*0.43/0.35*	4.37	*0.49/0.44*	3.89	*0.73/0.62*	2.4	2.1
**7a**	Au(I)	8.45	*1.52/1.29*	14.45	*1.07/1.00*	18.48	*0.92/0.87*	1.7	2.2
**7b**		18.62	*0.30/0.30*	36.31	*1.89/1.79*	60.26	*4.76/4.41*	1.9	3.2
**8a**	Pd(II)	2.68	*0.93/0.69*	5.25	*2.11/1.51*	5.96	*1.66/1.30*	2.0	2.2
**9a**	Cu(I)	0.072	*0.006/0.006*	0.84	*0.19/0.15*	1.04	*0.25/0.20*	11.6	14.3
**9b**		0.058	*0.002/0.002*	2.19	*0.45/0.38*	2.15	*0.94/0.65*	38.0	37.3
**10a**	Ag(I)	0.036	*0.009/0.007*	2.84	*0.19/0.18*	5.79	*1.93/1.45*	79.5	162.2
**Lc**	-	2.40	*0.39/0.34*	51.29	*17.26/12.92*	73.00	*1.02/1.00*	21.4	30.4
**Lb**	-	1.83	*0.32/0.27*	41.69	*14.68/10.86*	47.86	*11.70/9.40*	22.7	26.1
**IPr**	-	2.19	*1.00/0.69*	33.88	*2.09/1.97*	51.29	*5.73/5.15*	15.5	23.4
**SIPr**	-	5.05	*1.81/1.33*	54.95	*10.21/8.61*	105.52	*8.86/8.17*	10.9	20.9
**Etoposide**	-	0.12	*0.05/0.03*	1.11	*0.41/0.30*	1.90	*0.74/0.53*	9.0	15.4
**Cisplatin**	-	2.52	*0.75/0.58*	2.13	*0.99/0.68*	5.91	*2.29/1.65*	0.8	2.3

Supplementary Table S3. IC_{50} values with the standard deviation (SD) of the tested compound set against the murine breast cancer cell line CST, and its cisplatin resistant variant, CST-2/3. RR_CST: resistance ratio ($IC_{50\_CST\text{-}2/3}$ / IC_{50_CST}).

Compound	CST		CST-2/3		RR_CST
	IC_{50} [μM]	+SD/-SD	IC_{50} [μM]	+SD/-SD	
**1b**	2.81	*1.37/0.92*	4.92	*1.59/1.20*	1.8
**1d**	1.38	*0.36/0.29*	1.82	*0.73/0.52*	1.3
**2b**	1.01	*0.33/0.25*	2.17	*0.11/0.11*	2.2
**2e**	1.28	*0.07/0.07*	1.98	*0.25/0.23*	1.5
**3b**	22.91	*4.01/3.41*	25.12	*3.72/3.24*	1.1
**3d**	6.17	*0.53/0.49*	8.38	*0.33/0.32*	1.4
**4c**	1.10	*0.14/0.12*	1.35	*0.51/0.37*	1.2
**5a**	0.18	*0.03/0.03*	0.27	*0.06/0.05*	1.5
**6a**	0.53	*0.005/0.005*	0.88	*0.07/0.06*	1.7
**7a**	12.15	*5.41/3.74*	12.61	*5.92/4.03*	1.0
**8a**	1.52	*0.11/0.10*	2.00	*0.22/0.20*	1.3
**9a**	0.11	*0.01/0.01*	0.24	*0.07/0.06*	2.2
**10a**	0.092	*0.030/0.022*	0.218	*0.064/0.049*	2.4
**Lb**	4.95	*0.52/0.47*	9.69	*1.51/1.31*	2.0
**SIPr**	5.92	*0.63/0.57*	20.26	*0.42/0.42*	3.4
**Cisplatin**	0.15	*0.06/0.04*	0.82	*0.24/0.19*	5.5
**Carboplatin**	0.89	*0.21/0.17*	6.24	*2.02/1.53*	7.0
**Oxaliplatin**	0.41	*0.06/0.06*	3.84	*0.65/0.56*	9.3

Supplementary Table S4. IC_{50} values with the standard deviation (SD) of the tested compound set against the non-cancerous MSCL-1 mesenchymal stem cell like cell line. MSCL-1/Mes-Sa and MSCL1/CST are the ratios calculated from the IC_{50} values of the cell lines ($IC_{50\_MSCL\text{-}1}$/$IC_{50\_cancer\ line}$).

Compound	MSCL-1		MSCL-1 / Mes-Sa	MSCL-1 / CST
	IC_{50} [μM]	+SD/-SD		
**SIPR**	11.68	*3.34/2.60*	2.3	2.0
**9a**	0.81	*0.06/0.06*	11.2	7.3
**10a**	0.56	*0.09/0.08*	15.7	6.1
**5a**	1.15	*0.005/0.005*	2.7	6.5
**6a**	1.24	*0.04/0.04*	2.0	2.3
**7a**	25.71	*0.24/0.24*	3.0	2.1
**8a**	2.98	*0.05/0.05*	1.1	2.0
**Cisplatin**	17.03	*0.43/0.42*	6.8	113.6

<u>Supplementary Figure S2.</u> Structure of **1b*** and **1c***.

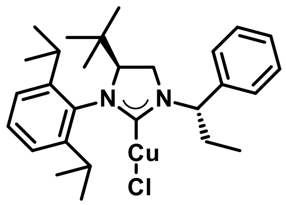

1b*

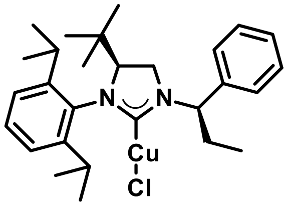

1c*
